# Comprehensive Review of Hydrogel-Mediated Strategies for Diabetic Wound Healing

**DOI:** 10.3390/ijms27093915

**Published:** 2026-04-28

**Authors:** Zihao Fan, Jie Li, Cheng Zhong, Dengzhuo Liu, Huiyan Fan, Litong Jiang, Guangwei Wang

**Affiliations:** 1 Hengyang Medical School, University of South China, Hengyang 421000, China; fzh552487926@outlook.com (Z.F.); lastxuan211@gmail.com (D.L.); 2 School of Medical Informatics and Engineering, Hunan University of Medicine, Huaihua 418000, Chinarakiraki556@outlook.com (C.Z.);; 3 School of Basic Medical Sciences, Hunan University of Medicine, Huaihua 418000, China

**Keywords:** hydrogel, diabetes, wound healing

## Abstract

Diabetic chronic wounds (particularly diabetic foot ulcers) are difficult to heal due to factors such as high glucose levels, infection, and inflammatory imbalance. In severe cases, they can lead to tissue necrosis and amputation. Hydrogel materials, as moist wound dressings, possess high water content, biocompatibility, and tunability, making them an important platform for promoting diabetic wound healing. In recent years, novel smart hydrogels have been developed to integrate multiple functions. They respond to abnormal stimuli in the wound microenvironment—such as acidic pH, high glucose levels, or excessive reactive oxygen species—to trigger the release of drugs, delivering on-demand antimicrobial, antioxidant, and anti-inflammatory effects. Simultaneously, they modulate immune responses (promoting macrophage polarization toward the M2 type) and stimulate angiogenesis, creating a microenvironment conducive to tissue regeneration. Some hydrogels incorporate antimicrobial agents, anti-biofilm components, or photothermal/photodynamic agents to effectively eliminate drug-resistant pathogens and control infections. Others serve as carriers for delivering stem cells and their exosomes, enhancing cell survival rates and releasing growth factors to accelerate wound healing. This review systematically summarizes recent advances in hydrogel strategies for diabetic wound treatment, focusing on stimulus-responsive hydrogels, antimicrobial and immune modulation mechanisms, pro-angiogenic and oxygen-supplying therapies, smart dressings and monitoring technologies, integration of stem cells and exosomes, as well as hydrogel injection, self-healing, and adhesion properties. Based on this, we analyze challenges and prospects for clinical translation of these strategies. Collectively, functionalized hydrogels hold promise as multifunctional therapeutic platforms for diabetic non-healing wounds. They offer a multi-pronged approach to disrupt the vicious cycle of “infection–inflammation–tissue destruction” thereby achieving more efficient wound healing.

## 1. Introduction

Diabetes-related chronic wounds, such as diabetic foot ulcers (DFU), represent one of the most severe complications for diabetic patients. Globally, approximately 15% to 25% of individuals with diabetes develop wounds that are difficult to heal [1]. These persistent wounds fail to resolve, with over half of cases potentially progressing to chronic ulcers. This significantly increases amputation rates and mortality, imposing a substantial healthcare burden [2]. Impaired wound healing in diabetes is largely driven by a complex pathological microenvironment. Persistent hyperglycemia directly damages tissues and promotes bacterial proliferation, leading to recurrent infection and biofilm formation. Wounds remain in a prolonged inflammatory state, with macrophages struggling to switch from the pro-inflammatory M1 phenotype to the pro-repair M2 phenotype, resulting in sustained high expression of inflammatory cytokines such as tumor necrosis factor-Alpha (TNF-α) and Interleukin-6 (IL-6) [3]. Additionally, advanced glycation end products and oxidative stress induced by high glucose levels cause vascular endothelial dysfunction, impeding neovascularization. Local ischemia and hypoxia further delay the healing process [4]. These pathological factors are not independent; rather, they interact in a self-reinforcing manner, whereby persistent oxidative stress helps maintain macrophages in a pro-inflammatory state, while unresolved inflammation further impairs angiogenic signaling and tissue repair. This mechanistic interplay helps explain why multifunctional hydrogels are needed to simultaneously target several pathological barriers rather than address only a single abnormality. Conventional treatments primarily rely on glycemic control, pressure relief, surgical debridement, and regular dressing changes. However, since these approaches cannot simultaneously address infection, inflammation, and inadequate blood supply, over half of DFU patients respond poorly to standard therapies [5]. Therefore, there is an urgent need to develop novel wound dressings with multiple therapeutic effects to provide comprehensive support for the healing of diabetic wounds.

Hydrogels are considered ideal wound dressing carriers due to their unique advantages. On one hand, their high water content and three-dimensional network structure help maintain a moist wound environment, absorb exudate, and isolate external contaminants, thereby promoting epithelial reconstruction and accelerated healing [6]. On the other hand, hydrogel networks can readily incorporate various drugs, growth factors, active nanomaterials, cells, or exosomes. Through rational chemical design, they can be engineered to respond to stimuli such as temperature, pH, glucose, reactive oxygen species (ROS), or enzymes, thereby enabling controlled and on-demand therapeutic release [7]. In recent years, extensive research has focused on “smart” hydrogel dressings that dynamically respond to the distinctive microenvironment of diabetic wounds. Importantly, this responsiveness should also be understood in relation to the different stages of wound healing. In the early inflammatory and infection-prone stage, diabetic wounds are typically characterized by acidic pH, elevated glucose, excessive ROS, high protease activity, and abundant exudate; under these conditions, hydrogels with relatively rapid swelling, partial degradation, or stimulus-triggered network loosening may facilitate the prompt release of antibacterial, anti-inflammatory, or antioxidative agents. During the proliferative stage, however, a more stable matrix and sustained release profile are generally more desirable to support fibroblast migration, angiogenesis, and extracellular matrix deposition. In the later remodeling stage, excessive persistence or continued high-dose release may interfere with tissue maturation, suggesting that gradual degradation and reduced therapeutic output may be more appropriate. Therefore, the design of smart hydrogels for diabetic wounds should not rely solely on responsiveness itself, but should aim to match swelling behavior, degradation kinetics, and drug release profiles to the evolving pathological needs of each healing stage. In addition to stimulus-responsive release, some hydrogels integrate photothermal, photodynamic, or enzyme-mediated oxygenation strategies to achieve dual intervention against local infection and hypoxia [8]. Functionalized hydrogels also serve as scaffolds for cells or exosomes, helping preserve biological activity and prolong their duration of action [9]. These multifunctional hydrogel systems have demonstrated significant therapeutic benefits in small-animal diabetic wound models, including accelerated wound closure, improved tissue regeneration, and reduced scar formation [10]. This review comprehensively examines the role of hydrogels in diabetic wound healing across key themes, including stimulus-responsive mechanisms, antibacterial and anti-inflammatory properties, immune modulation, angiogenesis promotion, oxygen delivery and release, smart monitoring, stem cell and exosome therapies, and hydrogel design optimization. It further discusses their prospects and challenges for clinical translation. Compared with previous reviews, the present review focuses not only on the functional diversity of hydrogels but also on how these systems address the key unmet needs in diabetic wound healing, particularly the dynamic and multifactorial pathological microenvironment and the challenges of clinical translation. Conceptually, this review is organized around the interplay between diabetic wound pathology and hydrogel function, highlighting how different hydrogel strategies can be matched to specific pathological barriers, healing stages, and translational demands.

## 2. Stimulus-Responsive Hydrogel

Stimulus-responsive hydrogels can be designed to respond to a variety of endogenous or exogenous triggers, including pH, glucose, reactive oxygen species (ROS), enzymes, temperature, light, and hypoxia. In this review, we primarily focus on pH-, glucose-, and ROS-responsive hydrogels, as these stimuli are most directly associated with the pathological microenvironment of diabetic wounds.

### 2.1. pH-Responsive Hydrogels

**Figure 1:**
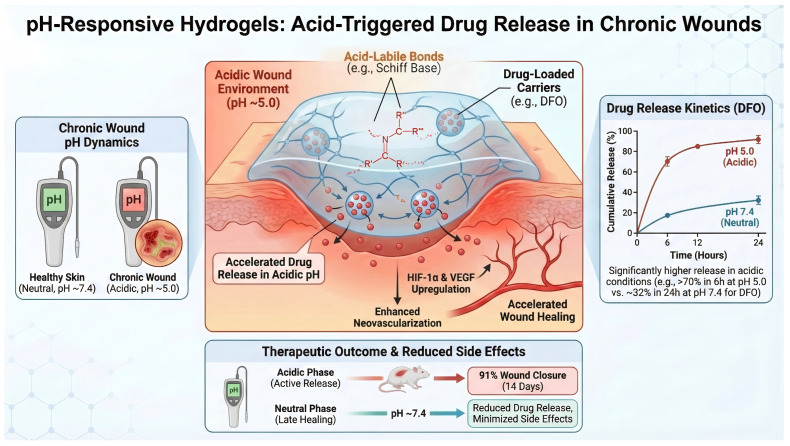
pH-Responsive Hydrogels: Acid-Triggered Drug Release in Chronic Wounds.

The pH of chronic wounds typically deviates from physiological neutrality and often becomes acidic during the stages of infection and persistent inflammation [11]. Hydrogels that trigger drug release in response to pH changes commonly incorporate acid-labile linkages, such as Schiff base (oxime) bonds or other dynamic covalent bonds, which remain relatively stable under neutral conditions but hydrolyze in acidic microenvironments. As a result, when these hydrogels are exposed to the acidified milieu of diabetic wounds, the network gradually loosens or degrades, thereby accelerating the release of encapsulated therapeutics. A representative example is a temperature/pH dual-responsive injectable hydrogel composed of chitosan oligosaccharide-pullulan (COS-F127) and aldehyde-modified hyaluronic acid, which undergoes in situ gelation at 37 °C and exhibits self-healing and adhesive properties [12]. When loaded with deferoxamine (DFO), a pro-angiogenic small molecule, this system released more than 70% of the drug within 6 h at pH 5.0, compared with only 32% over 24 h at pH 7.4. This pH-triggered release allowed DFO to act rapidly in hypoxic acidic wounds, upregulating HIF-1α and VEGF expression, promoting neovascularization, and accelerating wound closure in diabetic rats (91% closure by day 14) [13]. Thus, pH-responsive hydrogels can enhance local drug bioavailability at the lesion site and improve revascularization and tissue repair in difficult-to-heal wounds [14]. Compared with non-responsive hydrogels, this strategy offers better spatiotemporal control over drug delivery and may reduce premature drug leakage under near-physiological conditions. However, the therapeutic benefit of pH-responsiveness is also constrained by the dynamic nature of the wound microenvironment: diabetic wounds are not uniformly acidic throughout healing, and the pH may gradually shift toward neutrality during the later repair phase. Therefore, highly acid-sensitive systems may suffer from burst release in the early stage but insufficient sustained delivery afterward, which could limit long-term efficacy in chronic wounds [15]. In addition, pH-responsiveness alone does not address other pathological features of diabetic wounds, such as hyperglycemia, oxidative stress, or bacterial infection. From this perspective, recently reported multifunctional formulations that combine pH-triggered release with antioxidant, antibacterial, or metabolic regulatory functions may be more therapeutically advantageous than single-trigger platforms. Notably, as diabetic wounds may tend toward neutrality during the late healing phase, pH-responsive release can also help avoid unnecessary drug release at later stages, thereby potentially reducing side effects [15]. It should be noted that the cited study reported quantitative release profiles only under two pH conditions (pH 5.0 and pH 7.4). Therefore, Figure 1 is presented as a schematic comparison between acidic and near-neutral environments rather than a quantitative multi-pH release panel (Figure 1).

### 2.2. Glucose-Responsive Hydrogels

Persistent hyperglycemia is both a systemic factor contributing to poor wound healing in diabetes and a direct driver of local microenvironment deterioration. Elevated glucose levels suppress fibroblast and keratinocyte function while fostering bacterial growth that leads to infection. Glucose-responsive hydrogels operate in two ways: first, they “sense” elevated local glucose concentrations to trigger the release of insulin, hypoglycemic drugs, or other active agents, thereby directly reducing glucose levels at the wound site. Second, certain designs utilize glucose oxidase (GOx) to convert glucose into gluconic acid and hydrogen peroxide, consuming excess glucose while activating reactive oxygen species (ROS) response mechanisms [16]. A common glucose-responsive strategy involves incorporating phenylboronic acid (PBA) groups, which form reversible boronic acid esters with polyols (e.g., glucose’s ortho-diol). When local glucose concentration rises, glucose competitively binds PBA, causing dissociation of existing crosslinks and resulting in hydrogel network swelling or disintegration. However, the clinical translation of PBA-based systems may be limited by their relatively narrow glucose-responsive range and their sensitivity to pH fluctuations under physiological and wound conditions, both of which can affect response accuracy and stability. For instance, a study developed a hydrogel crosslinked with phenylboronic acid-modified chitosan and dopamine-modified oxidized hyaluronic acid. This hydrogel transitioned from a gel to a sol state within minutes in a 16 mM high-glucose solution, demonstrating sensitivity to hyperglycemic environments. Upon further loading with insulin, the hydrogel accelerated insulin release under hyperglycemic conditions, effectively reducing local blood glucose levels in diabetic wound models and accelerating wound closure (complete healing within 20 days) [17]. Similar principles apply to glucose-responsive crosslinking structures utilizing dynamic ester bonds, imidazopyrrole bonds, and others. Notably, integrating glucose responsiveness with oxidative stress treatment holds great promise. For instance, Advanced Science reported a multifunctional hydrogel combining “localized glucose control + antioxidant + photothermal” capabilities. Covalently grafted GOx enzymes in the wound oxidize glucose, reducing local sugar concentration while generating hydrogen peroxide. This peroxide is rapidly decomposed and cleared by co-loaded cerium oxide nanoparticles (CeO_2_) within the gel. This system achieves dual objectives of glucose reduction and ROS scavenging. Through synergistic anti-inflammatory effects, it accelerates the transition of macrophages in diabetic mouse wounds to the M2 phenotype, ultimately significantly enhancing healing rates [18]. Thus, glucose-responsive hydrogels demonstrate unique value in improving local metabolic environments and preventing sustained hyperglycemic damage, often synergizing with other functional modules to deliver comprehensive therapeutic outcomes [19].

### 2.3. ROS-Responsive Hydrogels

Elevated levels of ROS, such as hydrogen peroxide and superoxide anion, in chronic wounds damage cells and tissues, prolonging the inflammatory phase and hindering healing. Therefore, reducing oxidative stress is crucial for diabetic wound healing. ROS-related hydrogel strategies can be broadly divided into two categories. One type uses excessive ROS as a pathological trigger to induce hydrogel degradation, network loosening, or controlled drug release through ROS-cleavable linkages. The other type primarily functions as a ROS-scavenging platform by incorporating antioxidant molecules, enzymes, or nanozymes to directly neutralize free radicals and reduce oxidative stress. Although these two approaches may overlap in some multifunctional systems, they represent different therapeutic design principles [20]. For instance, one study encapsulated the anti-inflammatory Chinese herbal compound puerarin (PUE) and antimicrobial peptides within ROS-sensitive lipid nanoparticles, which were then loaded into methacrylate-modified gelatin hydrogels to create a composite dressing. This hydrogel accelerated drug release under excessive H_2_O_2_ conditions, significantly downregulating the expression of the pro-apoptotic protein caspase-3 in wound tissue, thereby reducing cell apoptosis and tissue damage. Simultaneously, it enhanced the activity of the Phosphoinositide 3-kinase (PI3K)/Protein Kinase B (Akt) signaling pathway in macrophages, promoting M2 polarization and the secretion of anti-inflammatory factors (such as interleukin-10 (IL-10) and Transforming Growth Factor Beta (TGF-β)). Results demonstrated that this hydrogel effectively scavenged excess ROS, suppressed inflammation, facilitated the transition of infected diabetic wounds to the proliferative phase, and accelerated healing [21]. Another strategy involves directly incorporating nanomaterials with enzyme-mimetic activity (nanocatalysts) into hydrogels, such as metal/metal oxide nanoparticles mimicking peroxidase, superoxide dismutase (SOD), and catalase (CAT) activities. Through cascade catalysis by nanoenzymes, harmful •O_2_^−^ and H_2_O_2_ can be converted into water and oxygen, simultaneously achieving dual antioxidant and oxygen-supplying effects. For instance, Sun et al. reported a sprayable “oxidative stress-driven oxygen-producing” hydrogel (OxyGel). Its cerium-based nanoenzymes (functionalized with azole phosphonate and tannic acid) possess dual SOD/CAT activity, continuously scavenging ROS and releasing oxygen upon application to wound surfaces. The synergistic action of this nanozyme hydrogel successfully reversed oxidative stress and hypoxia in diabetic wounds, significantly promoting macrophage M2 phenotype conversion while enhancing endothelial cell survival, migration, and neovascularization. In diabetic rat full-thickness skin defect and foot ulcer models, a single application of this hydrogel significantly accelerated wound healing within two weeks, achieving a much higher healing rate than the control group [22]. This innovation demonstrates that combining ROS responsiveness with oxygen production effectively remodels the wound microenvironment, offering an efficient and convenient therapeutic solution for chronic non-healing wounds [23].

Overall, stimulus-responsive hydrogels represent a rational strategy for diabetic wound management because they enable therapeutic release to be coupled with key pathological features of the wound microenvironment, such as acidic pH, hyperglycemia, and oxidative stress. Compared with non-responsive dressings, these systems offer improved spatiotemporal control and greater potential to modulate the wound environment in a targeted manner. However, each single-trigger strategy also has intrinsic limitations, since diabetic wounds are highly heterogeneous and dynamically evolve throughout healing. Importantly, these stimuli do not exist in isolation, but co-evolve across different healing phases: acidic pH and infection-related changes may dominate the early inflammatory stage, whereas persistent hyperglycemia, oxidative stress, and impaired angiogenesis may continue to interfere with later tissue repair. From this perspective, sequential or hierarchically responsive hydrogels that coordinate different functions over time may be more suitable than single-trigger systems for matching the dynamic trajectory of diabetic wound healing. At the same time, although many reported systems show promising outcomes in STZ-induced diabetic rodent models, these results should be interpreted cautiously, as such models do not fully recapitulate the complexity, chronicity, and heterogeneity of human diabetic wounds. Therefore, the most promising direction is likely not the optimization of one isolated response mechanism, but the development of multifunctional and stage-adaptive hydrogels that integrate several responsive elements to better address the complex and changing pathology of diabetic wounds.

## 3. Antimicrobial and Anti-Infective Strategies

Infection is one of the primary causes of persistent diabetic wounds. The hyperglycemic environment provides an ideal breeding ground for pathogens, making wounds highly susceptible to persistent bacterial infections. This leads to chronic inflammation and impairs the formation of new tissue. Therefore, effective antimicrobial action is fundamental to the treatment of diabetic wounds [24]. While traditional antibiotics remain an important therapeutic approach, their misuse can contribute to the proliferation of drug-resistant strains and immunosuppression. Hydrogel dressings achieve safe and efficient antimicrobial effects through multiple mechanisms:

### 3.1. Built-In Antimicrobial Drugs and Controlled-Release Systems

The most direct approach involves loading antibiotics or antimicrobial agents (e.g., levofloxacin, mupirocin) and bioactive substances with bacteriostatic effects (e.g., silver ions, chitosan, antimicrobial peptides) into the hydrogel matrix. The hydrogel provides a sustained-release carrier, maintaining effective drug concentrations at the wound site while minimizing systemic side effects. For example, research encapsulated the commonly used antibiotic ciprofloxacin (CIP) within a self-healing hydrogel. The resulting hydrogel dressing accelerated CIP release under the acidic, hyperglycemic, and high ROS conditions typical of diabetic wounds, achieving “on-demand” antibacterial activity [12]. In vivo experiments demonstrated that compared to the antibiotic-only group, this antimicrobial hydrogel more effectively cleared methicillin-resistant *Staphylococcus aureus* (MRSA) infections from wounds, reducing bacterial load while significantly alleviating tissue inflammatory responses [25]. Another example involves co-encapsulating broad-spectrum antimicrobial peptides (AMPs) with the statin drug pravastatin within silica and chitosan nanoparticles, then incorporating these into glucose/pH dual-responsive hydrogels. This approach enables simultaneous antimicrobial and pro-angiogenic effects. This composite hydrogel exhibited potent bactericidal activity against both MRSA and *Escherichia coli*. In an infectious diabetic foot ulcer rat model, it significantly reduced levels of inflammatory cytokines such as IL-6 and TNF-α while increasing anti-inflammatory factors like IL-10, indicating controlled infection alongside an improved inflammatory environment [26]. Furthermore, cationic polymers such as chitosan, quaternized chitosan, and ε-polylysine inherently possess antibacterial properties, killing or inhibiting bacterial growth through electrostatic interactions with bacterial cell membranes. These polymers are frequently incorporated into hydrogels as matrices to enhance antimicrobial performance. For example, a dual-crosslinked antimicrobial hydrogel employs network crosslinking of cationic polymers and phenylaldehyde polymers, loaded with the plant-derived active molecule asiaticoside. This hydrogel demonstrated outstanding antibacterial and anti-inflammatory effects in diabetic foot ulcer models, not only clearing wound infections but also reducing macrophage infiltration and pro-inflammatory factor expression while promoting angiogenesis [27]. Thus, through rational hydrogel design and drug combinations, broad-spectrum, highly effective local anti-infective therapy can be achieved [28].

### 3.2. Photothermal and Photodynamic Antibacterial Approaches

Non-antibiotic physical sterilization methods have garnered considerable attention as alternative strategies for combating drug-resistant bacteria and biofilms. Among these, photothermal therapy (PTT) utilizes photosensitive nanomaterials to convert near-infrared (NIR) light into localized heat, rapidly elevating wound temperatures to bactericidal levels. Photothermal antibacterial therapy offers several advantages, including strong controllability, broad-spectrum activity, and a low propensity to induce conventional drug resistance. Common photothermal agents include gold nanorods, graphene oxide, and polydopamine nanoparticles. For instance, polydopamine nanoparticles (PDANP) incorporated into the aforementioned multifunctional hydrogel not only served as carriers for DFO but also generated a photothermal effect under 808 nm laser irradiation, which, together with the intrinsic antibacterial activity of chitosan, enabled effective bacterial clearance in infected diabetic wounds [29]. Another study encapsulated gold nanorod-based nanostructures and glucose oxidase within ROS-responsive hydrogels, where the photothermal component mediated NIR-triggered sterilization while the cerium dioxide-related component scavenged excessive ROS and alleviated inflammation [30]. After treatment, both *Staphylococcus aureus* and *Escherichia coli* were effectively eliminated, accompanied by a reduction in M1 macrophages, an increase in M2 macrophages, and enhanced expression of pro-angiogenic factors such as VEGF, all of which accelerated wound healing [30]. In parallel, photodynamic therapy (PDT), which relies on photosensitizers to absorb light energy and generate bactericidal ROS, has also been incorporated into hydrogel systems. One representative design introduced reduced graphene oxide-fullerene composites into a dual-dynamic-bond crosslinked hydrogel, enabling simultaneous photothermal and photodynamic antibacterial effects under irradiation. The system also loaded pirfenidone (PFD), and the pH/glucose dual-responsive network accelerated PFD release, thereby suppressing excessive inflammation and promoting vascular regeneration [31]. Animal studies demonstrated that this photothermal/photodynamic synergistic hydrogel effectively reduced infection, decreased inflammatory mediators such as IL-1β and TNF-α, promoted collagen deposition and re-epithelialization, and significantly accelerated diabetic wound healing [32]. Compared with antibiotic-loaded or peptide-loaded antimicrobial hydrogels, phototherapy-integrated hydrogels provide an on-demand and localized antibacterial effect without directly increasing the selective pressure associated with conventional antibiotics. Nevertheless, their superiority is not absolute. PTT requires careful control of irradiation parameters to avoid collateral thermal injury to newly formed tissue, while PDT may be less effective in severely hypoxic diabetic wounds because ROS generation is oxygen-dependent. Moreover, the therapeutic outcome is highly dependent on light penetration depth, irradiation uniformity, and the availability of external equipment. These factors may substantially limit the practical applicability of photothermal therapy in large, deep, or irregular diabetic wounds, where effective light delivery can be more difficult to achieve [33]. Therefore, current evidence suggests that the most promising designs are not those relying on phototherapy alone, but rather multifunctional hydrogels that integrate photothermal/photodynamic sterilization with microenvironment modulation, such as antioxidant, anti-inflammatory, or glucose-regulating capabilities. Thus, integrating hydrogels with phototherapeutic techniques offers an antibiotic-free antimicrobial solution for diabetic non-healing wounds and demonstrates substantial potential against drug-resistant bacteria [33].

### 3.3. Other Emerging Antimicrobial Mechanisms

In addition to the antimicrobial strategies discussed above, a variety of other emerging approaches have been explored in hydrogel-based systems for infected diabetic wounds. Among these, metal ions and metal-based nanozymes, such as Cu^2+^, Zn^2+^, and transition-metal nanoclusters, have attracted considerable attention because of their ability to disrupt bacterial membranes or generate low levels of reactive species with antibacterial activity. However, their direct application may also increase the risk of cytotoxicity to host tissues. In this context, hydrogels can improve local biocompatibility and therapeutic efficacy by enabling controlled release and reducing peak exposure.

For example, one injectable hydrogel loaded with mesoporous zinc oxide exhibited pH-responsive Zn^2+^ release under the mildly acidic wound microenvironment, showing strong antibacterial activity against *Staphylococcus aureus*, while also alleviating inflammation and promoting angiogenesis [34]. Copper-based systems have shown similar potential. In one study, copper-peptide dimers were incorporated into ROS-responsive hydrogels, leading to effective antibacterial activity against MRSA and enhanced expression of angiogenesis-related genes in diabetic wound models [5]. Another emerging strategy involves incorporating enzymes capable of disrupting biofilms, such as lysozyme or proteases, into hydrogel systems. By degrading components of the biofilm matrix, these enzymes can weaken the physical barrier that protects embedded bacteria and thereby enhance the penetration and efficacy of subsequently released antimicrobial agents [35]. Overall, the versatility of hydrogels enables the integration of multiple antimicrobial mechanisms within a single platform, offering a promising strategy for managing the complex infections associated with diabetic wounds. With the continued development of new nanomaterials and bioactive agents, these multifunctional systems may provide increasingly effective approaches for local infection control [24].

Taken together, current antimicrobial hydrogel strategies for diabetic wounds have expanded well beyond simple local antibiotic delivery toward more integrated anti-infective platforms. Drug-loaded hydrogels remain attractive because of their relative simplicity and translational practicality, whereas photothermal/photodynamic systems and metal ion- or enzyme-assisted approaches provide complementary options for addressing drug-resistant bacteria and biofilms. Even so, none of these strategies is without limitations, as issues such as cytotoxicity, equipment dependence, release controllability, and formulation stability may affect real-world applicability. In light of these considerations, hydrogel systems that combine antimicrobial efficacy with broader microenvironmental regulation—such as anti-inflammatory, antioxidant, or pro-regenerative functions—are likely to represent the most valuable direction for future diabetic wound management.

## 4. Immune Regulation and Inflammation Control

Persistent inflammation in diabetic wounds is a key factor contributing to delayed healing. Under chronic hyperglycemic conditions, M1 macrophages within the wound become hyperactivated and continuously secrete pro-inflammatory mediators such as TNF-α, IL-1β, and IL-6. However, the transition to M2 macrophages (anti-inflammatory and pro-repair phenotype) is impaired [36]. Persistent M1 phenotype dominance leads to uncontrolled inflammation, disrupting the formation of new tissue. Concurrently, systemic and local immune dysfunction in diabetic patients (e.g., impaired T-cell function, weakened neutrophil chemotaxis) further hinders the transition into the proliferative remodeling phase. Therefore, restoring immune balance and alleviating chronic inflammation are crucial for promoting wound healing. Hydrogels offer multiple intervention strategies in this regard. By delivering anti-inflammatory drugs, immunomodulatory factors, or physical stimuli, they can induce macrophage phenotype shift from M1 to M2 and regulate cytokine networks, thereby creating an immune microenvironment conducive to healing [37].

### 4.1. Controlled Release of Anti-Inflammatory Drugs and Bioactive Molecules

Many anti-inflammatory drugs (e.g., glucocorticoids, NSAIDs) suppress inflammatory mediator production, but systemic administration may impair glycemic control or cause side effects. Localized controlled release via hydrogels enables anti-inflammatory effects at the wound site while minimizing systemic impact. For example, a “dual-responsive” hydrogel triggers insulin and celecoxib release upon high glucose and Matrix metallopeptidase 9 (MMP-9) activation. Celecoxib, a selective COX-2 inhibitor, downregulates inflammatory mediators like prostaglandins, thereby alleviating chronic wound inflammation. Results demonstrated significantly reduced wound TNF-α and IL-6 levels alongside accelerated granulation tissue formation in the hydrogel-treated group [38]. Beyond synthetic anti-inflammatory agents, plant extracts and active components from traditional Chinese medicine are also widely utilized. For instance, protocatechuic aldehyde (PA), a small molecule with anti-inflammatory and antioxidant properties, was combined with fish gelatin by Fu et al. to form an all-natural hydrogel. This formulation significantly increased the proportion of M2 macrophages in diabetic wounds, reduced pro-inflammatory factors like IL-1β, and promoted fresh collagen deposition and angiogenesis [39]. Asiaticoside, a triterpenoid compound derived from the traditional Chinese medicinal plant Centella asiatica, exhibits potent anti-inflammatory and wound-healing properties. When loaded into dual-crosslinked hydrogel, asiaticoside effectively suppressed macrophage infiltration in diabetic rat models, elevated IL-10 levels, and significantly accelerated wound closure [40]. Additionally, certain cytokines or bioactive peptides can be directly incorporated into hydrogels as immunomodulators. Examples include IL-10, TGF-β itself or its inducers, which directly supplement anti-inflammatory signaling; or thymosin and erythropoietin (EPO), which have demonstrated immunomodulatory and wound-healing effects. Although direct loading of protein-based factors faces challenges in stability and controlled release, the sustained release and protective functions of hydrogels can significantly prolong their effective duration [41]. In summary, local delivery of anti-inflammatory factors via hydrogels can effectively block persistent inflammatory signals in chronic wounds, creating conditions conducive to tissue repair [42].

### 4.2. Regulation of Macrophage Polarization

**Figure 2:**
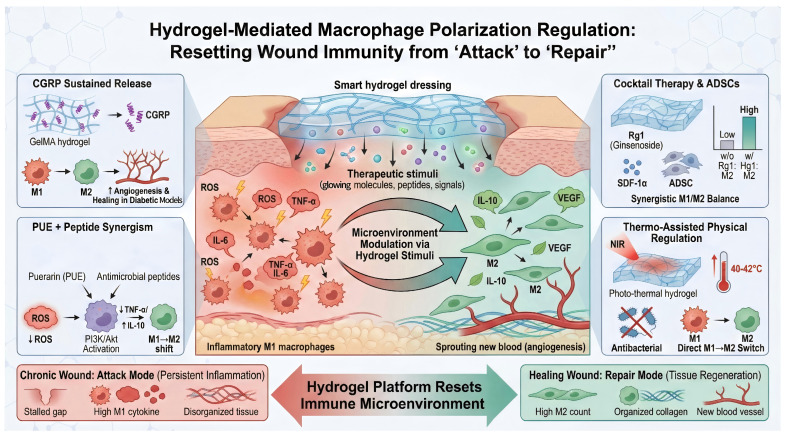
Hydrogel-Mediated Macrophage Polarization Regulation: Resetting Wound Immunity from ‘Attack’ to ‘Repair’.

Compared to direct drug administration, recent research has increasingly focused on guiding the intrinsic functional transition of immune cells within the body. The shift in macrophages from M1 to M2 represents the transition of a wound from the inflammatory phase to the proliferative phase, a process that can be modulated by microenvironmental cues. In general, M1-like macrophages are characterized by the production of pro-inflammatory mediators such as TNF-α, IL-1β, and IL-6, whereas M2-like macrophages are more commonly associated with anti-inflammatory and pro-repair factors such as IL-10, TGF-β, and VEGF. However, this M1/M2 classification should be regarded as a simplified framework, since macrophage phenotypes in vivo are highly heterogeneous and may exist along a dynamic functional spectrum rather than as two strictly separated states. Hydrogels can locally release stimuli that promote M2 polarization at the wound site, such as anti-inflammatory cytokines, microRNAs, drugs, or physical signals. A prime example is calcitonin gene-related peptide (CGRP), a neuropeptide found to be abnormally low in diabetic wounds. CGRP promotes macrophage M2 polarization and enhances endothelial cell proliferation. This effect is thought to be related to the ability of CGRP to modulate the inflammatory microenvironment, suppress excessive pro-inflammatory signaling, and promote a reparative immune phenotype that supports angiogenesis and tissue regeneration. Li et al. developed a CGRP-releasing gelatin methacrylate hydrogel that continuously delivers exogenous CGRP to the wound. Results demonstrated that local CGRP supplementation significantly increased the proportion of M2 macrophages in diabetic mouse wounds, markedly elevated neovascular density, and accelerated wound healing [43]. Similarly, the previously mentioned puerarin (PUE)-antimicrobial peptide composite hydrogel effectively suppressed macrophage M1 polarization and promoted the M2 phenotype by reducing ROS and activating the PI3K/Akt pathway. It simultaneously downregulated TNF-α and IL-6 while upregulating IL-10, achieving comprehensive optimization of the inflammatory cytokine profile [44]. A “cocktail therapy” hydrogel loaded with the immunomodulatory factors Rg1 (ginsenoside Rg1) and SDF-1α (stromal cell-derived factor-1), combined with ADSC stem cells, demonstrated in vitro that the Rg1-containing hydrogel significantly increased the M2 proportion in macrophages, whereas no significant M2 upregulation was observed without Rg1. Rg1 is known for its potent anti-inflammatory effects and ability to synergistically modulate M1/M2 balance [45]. This demonstrates that delivering cytokine inducers or active Chinese herbal molecules via hydrogels can effectively skew macrophage subpopulations and alleviate chronic wound inflammation. Notably, physical factors can also assist immune regulation. For instance, mild local heating (40–42 °C) has been demonstrated to facilitate M1-to-M2 conversion, inspiring the design of “heat-assisted” hydrogels. Thermosensitive hydrogels, for example, exhibit a slight temperature increase under light exposure, achieving dual therapeutic effects by not only sterilizing but also directly inducing macrophage phenotypic conversion [46]. However, this strategy requires careful control of irradiation intensity, exposure duration, and local temperature distribution, because excessive heating may cause collateral tissue injury, particularly at wound margins. In summary, diverse immune modulation strategies implemented through hydrogel platforms can reset the wound’s immune microenvironment, shifting it from an “attack mode” to a “repair mode” and paving the way for subsequent tissue regeneration [47] (Figure 2).

### 4.3. Controlling the “Inflammation-Aging” Axis

The pathological microenvironment of diabetic wounds may be further aggravated by cellular senescence and immune dysfunction. In these wounds, macrophages and other immune cells may exhibit immunosenescence-like features, leading to impaired responsiveness, defective phenotypic transition, and persistent low-grade inflammation. At the same time, chronic hyperglycemia, oxidative stress, and inflammatory signaling can promote premature senescence-like changes in wound-resident cells. These interconnected processes have been conceptualized as the “oxi-inflamm-aging” axis, in which oxidative stress, chronic inflammation, and cellular senescence mutually reinforce one another and contribute to impaired healing [48]. This framework is important because it suggests that merely suppressing inflammatory mediators may be insufficient; instead, more effective hydrogel-based interventions may need to simultaneously reduce oxidative stress, modulate inflammatory signaling, and restore immune-cell function. Recent studies have begun to explore such multifunctional approaches, for example, by incorporating antioxidant nanomaterials, autophagy-regulating components, or metabolic modulators into hydrogel platforms. Compared with conventional anti-inflammatory hydrogels, these systems may offer broader therapeutic benefit by targeting upstream mechanisms that sustain chronic wound pathology. However, their advantages should be interpreted cautiously, as the current evidence remains largely preclinical, and the relative contribution of antioxidation, senescence modulation, autophagy activation, and metabolic regulation has not yet been clearly distinguished. In addition, more biologically complex platforms may face greater translational challenges related to formulation complexity, delivery efficiency, and reproducibility. Overall, interventions targeting the oxi-inflamm-aging axis appear more aligned with the multifactorial nature of diabetic wounds than inflammation-only strategies, but further comparative and mechanistic studies are needed to determine which anti-aging or immune-restorative components are truly essential for clinical benefit [49].

From a broader perspective, immune-regulatory hydrogels represent a key strategy for correcting the dysregulated inflammatory microenvironment characteristic of diabetic wounds. Rather than acting solely as passive carriers of anti-inflammatory agents, these systems increasingly aim to actively reprogram immune responses, particularly by promoting macrophage polarization toward a pro-healing phenotype. Compared with direct anti-inflammatory drug delivery, approaches that modulate immune cell behavior or target upstream mechanisms such as the oxi-inflamm-aging axis may offer more sustained and physiologically relevant benefits. Nevertheless, current strategies still face important challenges, including incomplete understanding of immune heterogeneity in diabetic wounds, variability in therapeutic responses, and the complexity of integrating multiple immunomodulatory cues within a single platform. Consequently, future research should prioritize mechanistic clarity and comparative evaluation of different immunoregulatory approaches, with particular attention to identifying the minimal yet effective combinations required to achieve stable immune remodeling and improved clinical outcomes.

## 5. Strategies for Promoting Angiogenesis

Diabetic wounds heal poorly because of defective angiogenesis, which stems directly from the peripheral vascular lesions common in diabetes, resulting in inadequate blood supply to the wound area. Hyperglycemia and advanced glycation end products (AGEs) further exacerbate the problem by inhibiting endothelial cell proliferation and migration, thus impeding new capillary formation and causing local ischemia and hypoxia, which in turn limit tissue nutrient supply [50]. Therefore, promoting angiogenesis (vascularization) is essential for chronic wounds to enter the proliferative phase and initiate proper tissue regeneration. Hydrogels provide a highly effective platform for promoting angiogenesis: their soft, porous structure naturally favors endothelial cell and fibroblast infiltration, and they can also serve as efficient carriers for growth factors and cells, delivering localized, sustained pro-angiogenic signals.

### 5.1. Delivery of Angiogenic Factors

Growth factors such as VEGF, basic fibroblast growth factor (bFGF), and platelet-derived growth factor (PDGF) can directly stimulate vascular endothelial cell proliferation and neovascularization. However, their extremely short half-lives and tendency to diffuse limit the efficacy of systemic administration. The sustained-release properties of hydrogels make them highly suitable for the localized delivery of such growth factors [12]. For instance, a study incorporated recombinant human type I collagen (rhCOL1) and mesoporous zinc oxide into a self-healing injectable hydrogel. Collagen serves as an extracellular matrix component promoting cell adhesion and migration while also functioning as a binding scaffold for cytokines; ZnO provides antimicrobial and anti-inflammatory trace elements. This composite hydrogel significantly increased CD31-positive microvascular density in diabetic infected wound models. Collagen addition attracted endothelial cells and provided a footprint, while Zn^2+^ has been reported to induce expression of angiogenesis-related genes. The result was improved blood supply to the wound and significantly accelerated skin regeneration [39]. Additionally, some artificially designed pro-vascular small molecules or peptides have been integrated into hydrogels. For example, the C-terminal peptide of Insulin-like Growth Factor 1 (IGF-1C) has been demonstrated to possess pro-angiogenic effects similar to IGF-1. The multi-responsive hydrogel designed by Peng et al. incorporated a combined payload of IGF-1C peptide and DFO: IGF-1C enhances cell proliferation and metabolism, while DFO upregulates VEGF by stabilizing HIF-1α, synergistically promoting angiogenesis. In vivo experiments demonstrated that wounds treated with this hydrogel exhibited significantly higher vascular density and blood perfusion compared to controls [51]. DFO, a representative pro-angiogenic drug, prevents HIF-1α degradation by chelating iron and is widely used in ischemic diseases. In multiple diabetic wound models, DFO hydrogels consistently accelerated angiogenesis [52]. Similarly, SDF-1α, a bone marrow-derived stromal cell-derived factor, chemoattracts endothelial progenitor cells and stem cells to the wound site. Zeng et al. loaded nanoliposomes containing SDF-1α and Rg1 into an immunoneuroprotective hydrogel, resulting in enhanced peripheral vascularization and nerve regeneration around the wound [53]. Therefore, achieving sustained local release of pro-angiogenic factors via hydrogels represents one of the primary strategies for promoting vascularization in diabetic wounds. Notably, to enhance stability, these factors are often incorporated into hydrogels in bound forms (e.g., conjugated to collagen or polysaccharide matrices) or encapsulated forms (e.g., microspheres, nanocarriers), preventing rapid denaturation or diffusion-induced inactivation [54]. However, even with hydrogel-based delivery, growth factor therapy still faces important challenges, including burst release, limited long-term bioactivity, and variability in dose control within the protease-rich diabetic wound environment.

### 5.2. Microenvironment Improvement and Indirect Angiogenesis Promotion

Beyond direct delivery of pro-angiogenic molecules, hydrogels can also indirectly promote angiogenesis by enhancing the local microenvironment. For instance, the aforementioned oxygen-supplying hydrogel releases oxygen while scavenging ROS, transforming the wound from hypoxic to aerobic metabolism and thereby supporting endothelial cell function. Oxygen itself is also a crucial stimulator of angiogenesis: under oxygen-sufficient conditions, cells produce increased ATP, providing energy for new blood vessels; moreover, elevated local oxygen tension attracts vascular endothelium to migrate toward the hyperoxic zone. Thus, oxygen-supplying dressings promote the ingrowth of new capillaries into wounds to a certain extent [55]. Furthermore, following successful immune regulation, M2 macrophages secrete proangiogenic factors such as VEGF and PDGF. Thus, immune modulation and angiogenesis often complement each other. Experiments have demonstrated that increasing the proportion of M2 macrophages in wounds via hydrogels is accompanied by elevated levels of factors like VEGF and bFGF, as well as increased vascular density [56]. Furthermore, hydrogels provide a cellular adhesion scaffold, facilitating endothelial cell colonization and tubular structure formation at the wound site. For instance, hydrogels containing Extracellular Matrix (ECM) components like gelatin and collagen significantly promote endothelial cell adhesion and lumen formation; porous gel structures also enhance vascular ingrowth [37]. Certain metal elements, like copper, also stimulate angiogenesis, as copper ions serve as essential cofactors for the production and activation of angiogenic factors. Recent wound dressings have begun incorporating copper-containing components to enhance angiogenesis (e.g., copper nanoparticles or copper-modified peptide materials) [56]. In summary, angiogenesis is a multifactorial process, and hydrogels can promote it through multiple pathways, including factor provision, oxygen supply improvement, inflammation reduction, and scaffold guidance. In diabetic wound care, synergistic approaches are often required to effectively enhance vascularization, making multifunctional hydrogels particularly advantageous. For instance, a metal-polyphenol hybrid hydrogel leveraged a tannic acid-Fe^3+^ network for antimicrobial and anti-inflammatory effects, while Fe^3+^ promoted cell proliferation and angiogenesis, yielding favorable outcomes [48]. It is foreseeable that as understanding of the vascular biological mechanisms in diabetic wounds deepens, more novel pro-vascular factors (such as microRNAs and gene therapies) will be integrated with hydrogels, providing more powerful tools for reconstructing wound blood supply [49].

Overall, hydrogel-based angiogenic strategies for diabetic wounds can be broadly divided into two complementary categories: direct delivery of pro-vascular factors and indirect promotion of vascularization through microenvironmental improvement. The former provides a relatively straightforward means of enhancing endothelial activity, whereas the latter may offer broader and more sustained benefits by simultaneously correcting hypoxia, oxidative stress, inflammation, and matrix support. Nevertheless, the therapeutic efficacy of pro-angiogenic hydrogels is still constrained by issues such as the instability of growth factors, the complexity of angiogenic regulation, and the difficulty of reproducing robust vascularization in chronically impaired diabetic tissues. Importantly, increased expression of angiogenic markers or vessel density does not always indicate the formation of mature and functionally perfused vasculature, which is ultimately more relevant for durable wound repair. Therefore, the most promising direction is likely the development of multifunctional hydrogel platforms that combine vascular stimulation with immune regulation, oxygenation, and structural support, thereby addressing angiogenesis as part of an integrated wound-healing program rather than as an isolated target.

## 6. Supply of Oxygen and Nitric Oxide

### 6.1. Oxygen-Releasing Hydrogel

Insufficient wound oxygenation disturbs cellular metabolism and thus impairs wound repair, while diabetic patients suffer from aggravated hypoxia because of poor peripheral circulation. Traditional hyperbaric oxygen therapy is effective but has major drawbacks: it is expensive and not widely accessible. Therefore, locally oxygenating hydrogels have recently gained considerable attention. These hydrogels generally incorporate either chemical oxygen-generating components (e. g., calcium peroxide CaO_2_, sodium percarbonate, etc., which release H_2_O_2_ and decompose to yield O_2_) or physical oxygen-carrying agents (e. g., oxygen-rich microbubbles, perfluorinated compound emulsions, etc.). The oxygen-releasing microsphere-stem cell hydrogel described in the text is an excellent illustration of the strategy in question: by regulating the amount of immobilized peroxidase, the microspheres release oxygen from the hydrogel for three weeks, thereby markedly improving the survival rate and angiogenesis capacity of transplanted stem cells [55]. More importantly, in vivo experiments showed that the oxygen-supplying hydrogel strongly suppresses inflammatory factor expression TNF-α in wounds while upregulating growth factors like VEGF, accelerating ulcer healing in diabetic mice [5]. Another frontier approach involves enzyme-mediated oxygenation, utilizing nanozymes to convert endogenous H_2_O_2_ into O_2_ in situ within wounds. This approach avoids introducing exogenous chemical oxygen generators, offering a gentler and more sustained effect [21]. Results from nanozyme spray hydrogels indicate that slow oxygen release via nanozymes not only reverses wound hypoxia but also induces beneficial effects, including increased M2 macrophages and accelerated endothelial cell migration [57]. Compared to single-dose oxygenation methods like hyperbaric oxygen therapy, the sustained release of oxygen via hydrogels provides a more stable supply, maintaining physiological oxygen levels over extended periods to continuously stimulate all stages of wound healing [20]. Careful control of oxygen delivery rate and volume is essential to prevent new oxidative damage caused by oxygen excess. Consequently, some studies employ pH/ROS-responsive mechanisms to regulate oxygen delivery—accelerating oxygen production only when the wound environment becomes acidic or hydrogen peroxide accumulates, aligning with tissue repair demands [11]. In summary, locally oxygenating hydrogels serve as a valuable supplement to traditional oxygen therapy. By leveraging sustained-release principles to overcome rapid dissipation, they have demonstrated significant wound-healing effects in animal models [12].

### 6.2. Nitric Oxide (NO) Release

NO is a crucial gaseous signaling molecule with dual roles in wound healing: low concentrations promote vasodilation, increase local blood flow and nutrient delivery, and stimulate fibroblast proliferation and collagen deposition; NO also inhibits bacterial growth and modulates inflammation. However, NO gas itself diffuses rapidly and has an extremely short half-life, making its stable local delivery at the wound site a significant challenge. Hydrogels serve as carriers and release control mechanisms for NO [58]. However, the therapeutic effects of NO are highly dose-dependent, and the window between pro-healing and cytotoxic concentrations is relatively narrow. Accordingly, precise control of NO dosage and release kinetics is essential to maximize therapeutic benefit while minimizing potential tissue injury. One ingenious approach utilizes NO microbubbles: Chen et al. reported that trapping NO-filled microbubbles within hydrogels at the wound site prolongs local NO retention. This hydrogel demonstrated exceptional wound-healing efficacy in diabetic foot ulcer models: after just 11 days of treatment, wounds in the NO gel group achieved near-complete healing (residual area < 2%), significantly outperforming the control group [45]. Histological analysis revealed that NO gel-treated wounds exhibited thicker, more organized granulation tissue, longer re-epithelialization lengths, and significantly increased eNOS (endothelial nitric oxide synthase) expression. This indicates that NO released from the hydrogel not only acts directly but also activates a feedback pathway to stimulate endogenous NO production in endothelial cells, further promoting angiogenesis and tissue repair [39]. Furthermore, optimal NO levels inhibited apoptosis in fibroblasts and epithelial cells within the wound. TUNEL assays demonstrated markedly reduced apoptosis and increased Ki-67-positive areas (indicating proliferation) in the NO gel group [16]. Beyond NO microbubbles, certain NO donor compounds (e.g., the nitrosamines, divalent sulfides, and nitrates) can all be encapsulated in hydrogels to release NO in response to external stimuli such as light exposure or pH changes, and indeed, incorporation of nitrates into pH-responsive gels allows for a controlled, gradual release of NO in acidic wound environments, with excellent therapeutic results [31]. More importantly, NO-supplying hydrogels raise local NO levels to dilate blood vessels, improve blood flow, inhibit bacteria and inflammation, and promote cell proliferation, making them exceptionally well-suited for diabetic wounds, which are often complicated by microcirculatory disorders [33]. Therefore, future efforts should logically focus on fine-tuning NO release rates and total dosage to avoid cytotoxicity from excessive NO concentrations. Ultimately, the localized delivery of oxygen and NO—two small gas molecules—via hydrogels is an emerging and promising strategy for diabetic wound treatment, offering a direct way to improve wound microcirculation and accelerate healing [24].

In summary, hydrogels designed for oxygen and nitric oxide delivery provide a targeted approach to correcting hypoxia and microcirculatory dysfunction in diabetic wounds. Oxygen-releasing systems primarily enhance cellular metabolism, angiogenesis, and tissue viability, whereas nitric oxide-releasing hydrogels contribute additional benefits through vasodilation, antimicrobial activity, and modulation of inflammatory responses. Despite these advantages, precise control over gas release kinetics remains a critical challenge, as both insufficient and excessive delivery may compromise therapeutic outcomes. Moreover, differences in stability, delivery efficiency, and responsiveness among current formulations limit their consistency across complex wound environments. Consequently, future strategies should focus on developing responsive and finely tunable gas-delivery hydrogels, particularly those capable of coordinating oxygen and nitric oxide release with other therapeutic functions, such as antioxidant activity, immune modulation, and angiogenesis promotion, to achieve more comprehensive and clinically reliable wound repair.

## 7. Smart Dressings and Monitoring Technology

Due to recent advances in bioelectronics and sensing technology, “smart wound dressings” have become a clear and exciting new trend in diabetic wound management. Unlike conventional dressing changes, which depend on visual inspection and empirical judgment, smart dressings can continuously and objectively monitor wound pH, temperature, humidity, glucose levels, and inflammatory markers, then transmit the data to clinicians or patients to inform individualized treatment decisions. Hydrogels are particularly well-suited as the substrate for smart dressings because of their soft, biocompatible, and tunable properties, and thus embedding sensing elements or responsive materials into hydrogels enables the realization of an integrated “monitoring-feedback-treatment” function [59].

### 7.1. Adaptive and Visual Monitoring

The simplest smart feature of hydrogels is their intrinsic ability to respond to environmental changes, as clearly exemplified by pH-responsive hydrogels, which change color or fluorescence intensity in response to acidity/alkalinity shifts, thus serving directly and elegantly as visual wound pH indicators for infection or inflammation detection [60]. Similarly, glucose-responsive hydrogels can be scanned in vitro to monitor gel swelling or degradation, which correlates reliably with glucose levels in wound exudate (and indeed, several groups have designed hydrogels with embedded microsensors for this purpose). Despite their conceptual simplicity, these approaches have tremendous practical value for wound monitoring in resource-limited settings: highly transparent hydrogel patches have been reported that change color in response to glucose concentration changes while simultaneously providing hemostatic and antimicrobial functions, thereby enabling monitoring and treatment to occur concurrently [61].

### 7.2. Integrated Electronic Sensors

**Figure 3:**
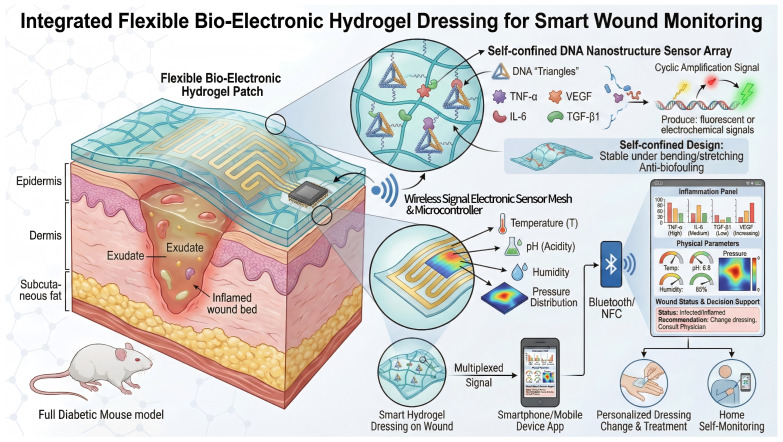
Integrated Flexible Bio-Electronic Hydrogel Dressing for Smart Wound Monitoring.

More recent smart dressings make excellent use of flexible electronics and nanotechnology to achieve highly reliable, multiparameter physiological sensing, as elegantly demonstrated by Zhao et al., who reported a flexible bioelectronic hydrogel patch employing self-confinement DNA nanostructure loops as biosensing elements. In this system, specific inflammatory proteins in wound exudate trigger cyclic amplification within DNA “triangular” structures, yielding easily detectable signals. Moreover, the self-confinement design of the DNA architecture ensures signal stability even under bending or stretching, and it also inherently resists biofouling. Signals are transmitted wirelessly, thus allowing real-time, simultaneous monitoring of several inflammation-related factors. (TNF-α, IL-6, TGF-β1, VEGF) alongside physical parameters like temperature and pH. In diabetic mouse models, it enables real-time, in situ tracking of healing status in both infected and non-infected wounds without interfering with the healing process. This integrated system combining hydrogels, biosensing, and wireless electronics represents a cutting-edge direction for smart wound dressings [62]. Furthermore, research has fused printable flexible electronics with hydrogels for continuous monitoring of wound moisture, pressure distribution, and other parameters, aiding in the assessment of pressure relief and rehabilitation progress [63]. These smart sensing hydrogel dressings hold promise for home self-monitoring: patients simply apply the dressing, and mobile devices retrieve wound condition data to enable personalized dressing changes and treatment decisions. In the future, incorporating additional sensor types—such as bacterial toxins and metabolic byproducts—will allow smart dressings to provide more comprehensive wound status profiles [64]. However, the practical reliability of these systems still depends on maintaining sensor calibration and signal stability over prolonged use in protein-rich, exudative wound environments, where biofouling and drift may gradually compromise measurement accuracy (Figure 3).

### 7.3. Feedback-Based Delivery and Closed-Loop Control

A truly ideal smart dressing should not only monitor wound status but also adjust treatment in response to the acquired information, which can be achieved by coupling sensing modules with controlled-release systems. At a basic level, stimulus-responsive hydrogels already provide a rudimentary form of closed-loop regulation, such as glucose-triggered insulin release or pH-triggered antibiotic release, whereas more advanced platforms integrate biosensors, flexible electronics, and actuators to modulate drug diffusion, local heating, or phototherapeutic activation in response to dynamic wound signals [65]. For chronic diabetic wounds, such systems are conceptually attractive because they may enable more precise and timely intervention than static dressings [66]. However, their clinical translation remains challenging, as these multifunctional platforms are often costly and technically complex, require stable signal accuracy and long-term performance in exudative and deformable wound environments, and may be affected by calibration drift during prolonged exposure to complex biological fluids. In addition, wireless data transmission in ambulatory settings raises practical concerns regarding power supply, device integration, and operational continuity, while large-scale manufacturing, sterilization, storage, and batch-to-batch reproducibility remain significant engineering barriers [67]. In addition, regulatory approval is likely to be more demanding than for conventional dressings because closed-loop smart hydrogels may be classified as combination products involving biomaterials, drugs, sensors, and wearable device components, which can create added regulatory complexity across major jurisdictions. Therefore, although closed-loop hydrogel dressings represent an important future direction, simpler and more robust semi-closed-loop systems may be more realistic for near-term clinical translation [68].

Smart dressings represent a clear, logical move toward “digital” and “personalized” diabetes wound care, since hydrogels offer an ideal platform that is both tissue-compatible and highly integrable, thus allowing sensing and control elements to coexist with biological tissues in a natural, functional manner. Although several smart hydrogel systems have shown encouraging results in animal models, clinical application is still some years away, and key challenges remain: sensor long-term stability, data accuracy, and dressing cost [67]. Nevertheless, smart hydrogel dressings remain a promising direction for future diabetic wound care. With continued advances in sensing reliability, system integration, and translational validation, such platforms may support more personalized and responsive wound management in the future [68].

## 8. Hydrogel Combining Stem Cells and Exosome Therapy

One key direction in regenerative medicine involves introducing regenerative-promoting cells (such as stem cells) or their secreted exosomes to wound sites to compensate for the insufficient intrinsic repair capacity in diabetic patients. Mesenchymal stem cells (MSCs) are widely recognized as beneficial for promoting wound healing due to their paracrine secretion of multiple growth factors and immunomodulatory factors. Specifically, MSC-derived exosomes (exosomes/extracellular vesicles, EVs) carry microRNAs, proteins, and other molecules, enabling MSC-like functions in a cell-free manner while avoiding the immunological and tumorigenic risks associated with cell transplantation [69]. However, both cells and exosomes face challenges when directly applied to wounds, including low survival rates and short functional durations: the enzymatic environment and hypoxic conditions of wounds rapidly kill transplanted cells or degrade exosomes. Hydrogels play a crucial role here, serving as three-dimensional scaffolds that protect and slowly release cellular products, thereby enhancing therapeutic efficacy [69].

### 8.1. Stem Cell Encapsulation and Survival Improvement

**Figure 4:**
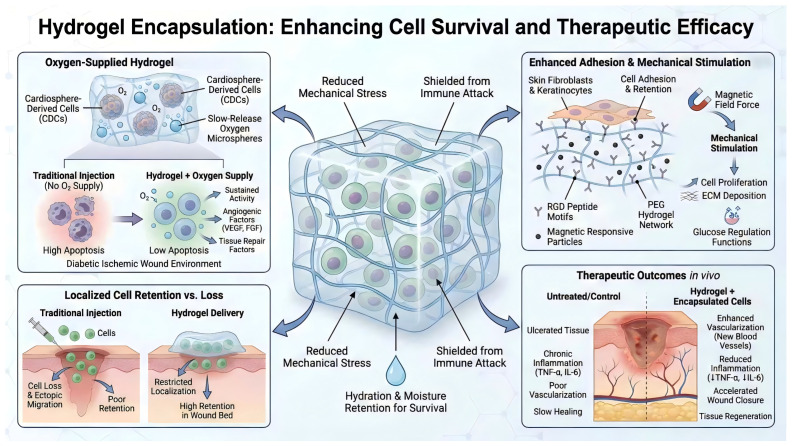
Hydrogel Encapsulation: Enhancing Cell Survival and Therapeutic Efficacy.

Encapsulating live cells within hydrogels provides a biomimetic three-dimensional culture environment, reducing cellular exposure to mechanical stress and immune attacks. Simultaneously, the gel’s moisture-retaining properties favor cell survival [39]. Chen et al. incorporated cardiac progenitor cells (CDCs) with slow-release oxygen microspheres into hydrogels, creating an “oxygen-supplying” stem cell transplantation system. Due to the hydrogel’s sustained oxygen release, transplanted CDCs exhibited significantly reduced apoptosis and maintained viability within diabetic ischemic wound environments. Consequently, these surviving stem cells secreted abundant angiogenic and tissue-repairing factors. In animal models, significantly enhanced vascularization of ulcer tissues, downregulation of inflammatory factors, and markedly accelerated wound healing were observed [55]. Beyond oxygen delivery, hydrogels can also enhance cell adhesion and retention within the matrix by incorporating cell adhesion peptides such as Arginine-Glycine-Aspartic acid (RGD). For instance, a study in Adv. Mater. designed a PEG hydrogel containing RGD motifs and magnetically responsive particles to load skin fibroblasts and keratinocytes. Mechanical stimulation via an external magnetic field significantly promoted cell proliferation, ECM deposition, and glucose regulation-related functions. This demonstrates that the three-dimensional scaffold environment provided by hydrogels, combined with appropriate stimulation, can activate the regenerative potential of cells [51]. Furthermore, certain hydrogels inherently incorporate nutrients or protective agents essential for cell survival, such as serum albumin or gelatin, thereby sustaining cellular function. In stem cell therapy, hydrogels effectively overcome issues associated with traditional cell injection—including wound leakage and ectopic migration—by confining cells locally and enhancing the microenvironment, significantly improving the feasibility of stem cell therapies [56] (Figure 4).

### 8.2. Exosome and Secretory Product Delivery

Compared to cells, MSC exosomes are smaller in size (30–150 nm), easier to store, and exhibit low immunogenicity, making them a rising star in diabetic wound therapy in recent years. However, after injection into wounds, exosomes are prone to lymphatic clearance or dilution by diffusion, making it difficult to maintain effective concentrations. Hydrogels can serve as “reservoirs” for exosomes, enabling sustained release [70]. Xu et al. developed a dual-physically crosslinked viscoelastic hydrogel (PSiW) uniformly loaded with human foreskin-derived MSC exosomes (FM-EVs). This hydrogel exhibits self-healing adhesion and antimicrobial properties, conforming to wounds while gradually releasing exosomes. Animal studies demonstrated that the exosome-hydrogel combination significantly outperformed exosome monotherapy in diabetic wounds: it modulated macrophage polarization (increasing M2, decreasing M1), promoted angiogenesis, and comprehensively improved the wound microenvironment. At 14 days, treated wounds were nearly fully healed, whereas free-exosome-treated wounds remained unhealed. This outcome demonstrates that hydrogels enable exosomes to “reside” locally at the wound site and exert their full effects [71]. Similarly, exosome-loaded hydrogels can synergize with other therapies. Wu et al. proposed a “dual-mode” strategy: topically applying responsive hydrogels while intravenously administering MSC exosomes to regulate systemic hyperglycemia. This combined approach yielded remarkable results in mice, with wound healing significantly outpacing monotherapy and demonstrating superior long-term efficacy. Although intravenous exosome administration constitutes systemic therapy, it underscores the concept of synergistic interaction between local hydrogels and systemic interventions: hydrogels improve the local environment (antibacterial, anti-inflammatory, drug release), while exosomes enhance systemic metabolism (hypoglycemia, immunomodulation). This complementary dual approach provides more comprehensive treatment [72]. This holds clinical relevance, as relying solely on local wound management may fail to overcome the systemic effects of diabetic conditions. Future stem cell/exosome therapies will likely adopt a “combination approach”: prioritizing local hydrogels supplemented by systemic treatments, or integrating stem cells, gene carriers, etc., into hydrogels for “one-stop” delivery [73].

### 8.3. Tissue-Engineered Skin and Biostructures

Beyond embedding cells/exosomes in hydrogels for direct wound application, another approach involves using hydrogels to construct tissue-engineered skin substitutes that cover wounds and promote full-thickness skin regeneration. For instance, MSCs or stem cells are cultured on biomimetic hydrogel matrices to form tissue sheets mimicking dermal-epidermal architecture, which are then transplanted onto diabetic ulcers. This approach resembles artificial skin concepts but incorporates stem cell activity. The hydrogel mimics the ECM and provides mechanical support. While preliminary attempts exist, research remains needed on preventing recurrence of lesions post-transplantation for chronic wounds [45].

Overall, stem cell and exosome therapies offer means to promote regeneration at the source for difficult-to-heal wounds, with hydrogels serving as critical carriers for their successful application. Whether enhancing cell/exosome survival, preserving their functionality, or directing their action, hydrogels play an indispensable role. Looking ahead, as our understanding of MSC exosome mechanisms deepens, we may achieve more precise regulation by targeting specific miRNA-or protein-enriched exosomes to wounds. Technologies like 3D printing could also produce cell-laden hydrogel scaffolds that more closely resemble native skin tissue for transplantation. However, the clinical translation of cell- and exosome-based hydrogel systems still faces major manufacturing and regulatory challenges. In particular, exosome therapies require GMP-compliant isolation and purification processes, while the cargo composition of exosomes may vary substantially between donors, cell sources, and production batches, which complicates product standardization. In addition, the lack of widely accepted potency assays remains a major obstacle for quality control, comparability, and regulatory approval of these biologically complex products. It is foreseeable that “hydrogel + biotherapy” will become a vital component of regenerative medicine for diabetic wounds, delivering substantial healing breakthroughs for patients [24].

## 9. Hydrogel Design Enhancements: Injectable, Self-Healing, and Adhesive Properties

An ideal diabetic wound dressing must not only provide therapeutic functions but also satisfy practical requirements related to physical performance and ease of use in real clinical settings. In this section, we focus specifically on engineering design properties of hydrogels—such as injectability, self-healing, adhesion, and mechanical balance—that influence dressing conformability, retention, durability, and usability, rather than on the therapeutic mechanisms discussed in earlier sections. Given that wounds such as diabetic foot ulcers are often irregular in shape and located in mechanically active areas, hydrogel dressings must conform closely to the wound surface and resist detachment or tearing during daily movement. Furthermore, because chronic wound treatment often requires prolonged management and repeated dressing changes, self-repairing or durable hydrogel systems may reduce both patient discomfort and treatment burden [74].

### 9.1. In Situ Molding and Shape Adaptation

Injection-molded hydrogels undergo sol–gel phase transitions, thereby allowing liquid precursors to solidify in situ at the wound site and thus fill wounds of any shape while conforming perfectly to the wound surface. A very clear and elegant example is the COS-pullulan/HA hydrogel, which has pronounced shear-thinning properties due to dynamic covalent bonds, making it ideal for injection into wounds, where it rapidly solidifies into stable gels upon retention. Therefore, this approach is exceptionally well-suited for deep, crater-like, or irregular ulcers, as it fills dead spaces effectively and minimizes fluid accumulation and residual infection foci [40]. A representative example is a glucose- and MMP-9-dual-responsive hydrogel with a temperature-sensitive self-adaptive shape, which can conform to irregular wound surfaces and enable controlled drug release in the diabetic wound microenvironment [75]. More recently, researchers have developed thermoshrinkable hydrogel dressings via 4D printing technology, which automatically contract under body-temperature stimulation and actively draw wound edges together to facilitate closure. This behavior is generally achieved through shape-memory or thermoresponsive polymer networks that store a temporary configuration during fabrication and recover a programmed structure upon thermal activation. In this context, the therapeutic value of 4D-printed hydrogels depends not only on their ability to shrink but also on how precisely the magnitude and distribution of contraction forces can be matched to wound geometry, since excessive or uneven contraction may compromise tissue adaptation or mechanical stability. Lu et al. reported that such 4D-printed hydrogels markedly accelerated wound contraction and healing in MRSA-infected diabetic rat ulcers [76], supporting the potential of shape-adaptive hydrogel systems to improve conformability and wound closure [77].

### 9.2. Self–Healing and Durability

Self-healing hydrogels can restore their integrity after damage by dynamically reshaping their structure through dynamic bonds. This proves highly practical for wounds requiring frequent dressing changes or subjected to mechanical stress, preventing dressing failure or performance degradation. Designs incorporating dual networks or multiple dynamic bonds (hydrogels) achieve exceptional self-healing capabilities. For instance, a triple-network hydrogel based on natural polysaccharides exhibits remarkable self-healing efficiency through hydrogen bonds and hydrophobic interactions, fully repairing within minutes after re-contact following tearing [78]. Similarly, Schiff base-crosslinked hydrogels often demonstrate rapid self-healing due to the reversible cleavage and re-formation of oxime bonds. Research by Pan et al. indicates that Schiff base-type self-healing hydrogels are particularly suited for “active” wounds: they not only exhibit strong adhesion (through covalent bonding between Schiff bases and tissue amines) but also dynamically crosslink in acidic inflammatory environments, facilitating pH-responsive drug release. Their PFD photothermal hydrogel achieves outstanding self-healing and reusable adhesion through dual crosslinking with Schiff bases and borate esters. Rat activity model tests demonstrated that this hydrogel resists rupture and detachment even under frequent joint flexion [79]. Another advantage of self-healing is extended dressing lifespan: patients need not replace entire dressings daily; localized damage can self-repair, or drugs can be replenished locally, reducing costs and wound interference. Future designs integrating micro-puncture or microfluidics could enable multi-dose drug-loading hydrogel dressings, realizing the concept of “single application, multiple drug delivery” [80].

### 9.3. Strong Adhesion and Fixation

Adhesive properties are particularly important for hydrogel dressings in diabetic wounds, especially diabetic foot ulcers, which often occur at high-friction and high-stress sites such as the plantar surface. In such settings, inadequate adhesion can lead to dressing displacement, shortened retention, and unstable therapeutic delivery, whereas excessive adhesion may damage fragile neo-tissue during removal. Therefore, the goal of adhesive hydrogel design is not to maximize adhesion alone, but to achieve an appropriate balance between stable fixation and atraumatic removal. In practical terms, this balance may depend on tuning interfacial bonding strength, hydration state, and dynamic reversibility, so that the dressing can remain stable during exudation and motion but still be removed with minimal disruption to fragile neo-tissue. Current adhesive hydrogels generally rely on several major strategies, including catechol- or dopamine-mediated interfacial bonding, dynamic covalent interactions such as Schiff base or boronate chemistry, and physical interpenetration or electrostatic interactions with the wound surface [81]. Among these, catechol-based systems often provide rapid and robust wet-tissue adhesion, which is advantageous for irregular and exudative wounds, but overly strong interfacial binding may complicate dressing replacement. By contrast, dynamically crosslinked hydrogels are increasingly attractive because they can combine moderate adhesion with self-healing, injectability, and structural adaptability, thereby better accommodating the moist and mechanically active environment of diabetic wounds [82]. Sprayable hydrogels represent another useful approach, particularly for irregular or large wound areas, because they can form conformal coatings in situ; however, their retention under repeated motion may be less reliable than that of more cohesive adhesive systems [83]. Overall, the most promising adhesive hydrogels for diabetic wounds appear to be those that integrate durable wet adhesion with reversible network dynamics and additional therapeutic functions, rather than those optimized solely for maximal adhesive strength. Future studies should place greater emphasis on clinically relevant comparisons of fixation stability, removal-associated tissue injury, and performance under exudative and high-motion conditions [52].

### 9.4. Mechanical Properties and Balance

While emphasizing softness and conformability, hydrogels must also possess sufficient mechanical strength to protect wounds from external forces. Multi-crosslinked networks (such as dual-network hydrogels) often exhibit high toughness and resistance to tearing. For instance, Gastrodia polysaccharide-based triple-network hydrogels demonstrate excellent mechanical properties capable of cushioning external impacts [12]. Magnetically responsive hydrogels can dynamically harden or soften under external magnetic fields, enabling tailored mechanical stimulation or protection for wounds. Shape-memory hydrogels temporarily deform upon impact and revert to their original shape afterward, offering another protective mechanism. All these optimizations aim to make hydrogels both soft and resilient, meeting the stringent demands of clinical applications. Nature magazine commentary emphasizes that inexpensive, easily prepared hydrogel dressings are particularly crucial in resource-limited regions. Consequently, researchers are reducing costs by simplifying crosslinking reactions and utilizing natural materials (gelatin, alginates, etc.). Because the present effort is directed at developing 3D printing and mass production techniques to speed up the practical application of advanced hydrogels, it is very logical and well-founded to anticipate that next-generation hydrogel dressings for diabetic wounds will be multifunctional while offering a markedly improved user experience: clinicians and patients will be able to apply injectable/sprayable hydrogels as easily as ointment, the dressings will adhere firmly to the wound, deliver therapeutics continuously, monitor wound status in real time, and give immediate feedback, therefore merging therapy and management into one seamless step [24].

Overall, advances in hydrogel design have increasingly emphasized not only therapeutic functionality but also the practical requirements of diabetic wound dressings in real clinical settings. Injectable and in situ forming systems improve conformability to irregular wounds, whereas self-healing and adhesive designs enhance retention, durability, and tolerance to repeated mechanical stress during daily activity. At the same time, optimization of mechanical properties remains essential because hydrogel dressings must balance softness and adaptability with sufficient toughness to protect the wound and maintain structural integrity. These design parameters should not be considered independently, since excessive emphasis on one property may compromise others, such as atraumatic removal, permeability, or patient comfort. Therefore, the most promising hydrogel platforms are likely to be those that integrate injectability, self-healing capacity, appropriate adhesion, and mechanical robustness within a multifunctional yet practically applicable dressing system. Future progress will depend not only on material innovation but also on simplifying fabrication, reducing cost, and improving manufacturability to facilitate broader clinical translation.

## 10. Challenges and Prospects in Clinical Translation

Although hydrogels have great potential for diabetic wound healing, there are several important obstacles to overcome before they can be successfully translated from the laboratory to the clinic.

### 10.1. Safety and Biocompatibility

Because many novel hydrogels incorporate nanomaterials, synthetic polymers, and active pharmaceutical ingredients, it is essential to conduct systematic, rigorous evaluations of their long-term biological safety. Specifically, one must address the degradation and clearance pathways of metallic nanoparticles and nanoenzymes in tissues, and determine whether they could accumulate to toxic levels or trigger allergic reactions [84]. Similarly, certain dynamic covalent crosslinkers (e. g., aldehyde groups, boronic acids) are cytotoxic at high concentrations, so a key challenge is to achieve complete gel polymerization without releasing residual harmful monomers. Therefore, the proper approach is to validate these aspects through well-designed animal and clinical trials. Fortunately, most hydrogels reported in the literature already show excellent biocompatibility in cell and mouse studies. Since there is no evidence of significant inflammation or toxicity (but longer-term, large-animal data are still needed for definitive confirmation), and because regulatory approval for devices containing cellular/genetic materials or high-risk components is extremely rigorous, material safety must be rigorously established before translation.

### 10.2. Efficacy and Reproducibility

Animal models (mice, rabbits) exhibit significant differences from human wounds: mouse skin healing relies primarily on contraction with high metabolic activity, whereas human diabetic foot often involves vascular and neurological complications with severely impaired local circulation and sensation. Thus, hydrogel therapies effective in animals may not work in humans, or may require longer durations and multiple applications to demonstrate efficacy. Furthermore, wounds vary greatly among patients—differing in bacterial profiles, necrosis severity, and systemic conditions—raising questions about the applicability of a single formulation to all cases. For instance, hydrogels targeting Gram-positive bacteria may be less effective on wounds dominated by Gram-negative infections. Future products may thus require tailored formulations (e.g., distinct formulations for staphylococcal-dominant and Pseudomonas-dominant infections). Simultaneously, clinical trials must demonstrate statistically significant accelerated healing effects in larger sample sizes. Currently, most products entering clinical trials feature relatively simple combinations (e.g., hydrogels containing growth factors or antimicrobial agents), while multifunctional advanced gels lack clinical validation data.

### 10.3. Ease of Use and Patient Compliance

Most diabetic wound patients are elderly, requiring dressings that are simple and straightforward to apply. Complex procedures—such as those involving specialized light therapy devices or magnetic field stimulation—may prove difficult to implement in real-world settings. Even smart dressings face compliance challenges if patients must connect devices or calibrate parameters themselves. Therefore, streamlining the application process is crucial. Fortunately, many innovations focus on this area. For example, spray-on hydrogels can cover wounds as easily as applying medication, with a single application lasting several days or even up to two weeks. While 4D-printed personalized patches require scanning and printing equipment for production, they could be centrally manufactured by hospitals for patients to apply at home. Smart monitoring patches aim to operate automatically upon application, minimizing manual intervention. The overarching trend is toward disposable, multifunctional, and portable solutions, hiding complex technology behind simple user experiences to enhance patient acceptance and compliance.

### 10.4. Cost and Mass Production

Highly complex multi-component hydrogels generally require laborious preparation procedures and therefore have high production costs, which is why reducing costs while preserving performance and achieving scalable production remain major challenges in engineering translation. Specifically, rare metals or expensive biological products that are cost-negligible at experimental scales can become extremely costly at industrial batch levels. Hence, one natural direction is to seek more economical material substitutes: for example, silver instead of gold nanorods, or microbial fermentation for growth factors instead of recombinant protein production. Similarly, although 3D printing and microfluidic fabrication offer exceptional control over structure, their low yields necessitate intelligent integration with traditional industrial methods. Because batch cross-linking reactions and freeze-drying molding can both improve efficiency, regulatory compliance ultimately rests on product consistency and controllability: the composition and properties of hydrogels must be consistent across batches, hence the need for rigorous quality control and well-standardized processes. Many studies use natural extracts (e. g., plant polysaccharides, proteins) which inherently exhibit batch-to-batch variability. Therefore, when such materials are used in medical dressings, special attention must be given to their standardization to meet regulatory requirements.

In addition to general issues of safety, cost, and manufacturability, the clinical translation of multifunctional hydrogel dressings also depends on a clear regulatory strategy. For products that combine biomaterials with drugs, biologics, sensors, or nanomaterials, regulatory classification may vary according to the primary mode of action and product risk profile; in the U.S. context, some products may follow a 510(k) pathway if an appropriate predicate exists, whereas more complex or higher-risk products may require De Novo classification or PMA-level review rather than a simplified device pathway. For nanomaterial-containing systems, there is currently no ICH guideline specific to wound dressings, but relevant ICH quality and risk-management principles remain important for product characterization, manufacturing control, and safety evaluation. In addition, pivotal clinical trials for diabetic foot ulcer products should use clinically meaningful endpoints. Complete wound closure is generally considered a more robust endpoint for confirmatory studies, whereas percentage area reduction may be more appropriate for exploratory or early-phase evaluation and does not necessarily establish definitive clinical benefit. Therefore, future hydrogel translation will require not only material optimization, but also early alignment of product classification, CMC/quality strategy, and trial endpoint design.

Because the successful adoption of novel hydrogel dressings depends on their integration into existing wound care systems, it is essential to train physicians and nurses adequately on the advantages of the new products and their proper application techniques. Traditional wound management approaches (e.g., pressure-relieving shoes, antibiotics, and negative pressure therapy all remain effective, and therefore new hydrogels should be incorporated as part of a complete, rational treatment plan rather than replacing all existing methods. Specifically, diabetic foot ulcers with severe osteomyelitis still require surgical debridement combined with antibiotics, with hydrogels used as an adjunct to promote healing. Hence, positioning these products appropriately and developing sound clinical guidelines are major challenges. More importantly, clinical trials must demonstrate superiority over standard therapies to convince healthcare systems to adopt the new products. Physicians tend to approach new technologies, especially high-tech smart dressings, with caution, so clear, robust evidence showing improved healing rates or shorter healing times is necessary to actually change established practice.

Even with all these headaches, one thing’s crystal clear in every clinic and research lab I have seen: diabetic wounds need real game-changers, and fast. Hydrogels feel like the dark horse that might actually close the gap that has haunted us for decades. Look at what’s already happening. That 4D patch they have been testing just slipped into human trials, and the first numbers coming back are quietly thrilling—wounds closing way quicker, no nasty surprises on the side-effect front. Same story with those silver-laced antimicrobial gels; they are already sitting on pharmacy shelves, quietly performing the heavy lifting on ulcers that used to laugh at everything else. Give it another five or ten years, and I suspect we will be spoiled for choice. More of these clever, multitasking hydrogels will roll out, handing doctors fresh weapons they never had before. The whole approach to diabetic foot care is already tilting toward something bigger than one specialty—endocrinologists locking down blood sugar, orthopedic folks sorting out pressure points and bone issues, while the bioengineered dressings race ahead on the actual tissue repair. Patients are the ones who will feel it first: fewer midnight fears about amputation, legs healing in weeks instead of months, and mornings where getting out of bed does not feel like a negotiation. None of this will happen by magic, of course. It is going to take the labs, the bedside teams, and the companies all rowing in the same direction. Still, every new paper on hydrogels leaves you with that same restless energy—like the future just leaned in and whispered that it is possible. Truth is, diabetic foot ulcers are starting to look less like an unbreakable curse and more like a problem we are finally learning how to outsmart. And these gels? They are right in the middle of the fight, changing the odds one layer at a time.

## 11. Conclusions

Hydrogels have emerged as highly promising platforms for the management of diabetic wounds because their unique physicochemical properties enable the integration of multiple therapeutic functions within a single dressing system, including antimicrobial activity, antioxidant effects, immunomodulation, promotion of angiogenesis, oxygen delivery, and cell or bioactive factor transport (a summary of these strategies is provided in Appendix A: Table A1). Importantly, evidence from animal studies and early clinical investigations suggests that hydrogel-based dressings can modulate the hostile wound microenvironment, disrupt the persistent cycle of inflammation and infection, and thereby accelerate wound closure and tissue regeneration. These advances also align with the broader trend toward precision medicine, as recent hydrogel systems increasingly incorporate stimuli-responsive drug release, real-time wound monitoring, and programmable regulation of the healing process. In addition, properties such as tissue adhesion, self-healing, injectability, and shape-memory behavior further enhance their practical value in complex wound care settings.

However, despite these advances, a key unresolved issue is how to design the most appropriate hydrogel for the specific pathological features of diabetic wounds. There is unlikely to be a universally optimal formulation, because diabetic wounds are characterized by persistent inflammation, bacterial infection, oxidative stress, impaired angiogenesis, excessive exudation, hypoxia, and, particularly in diabetic foot ulcers, repeated mechanical stress and irregular wound geometry. These pathological features should directly inform hydrogel design parameters. For example, sufficient mechanical strength is required to maintain structural integrity and wound coverage under moist and mechanically challenging conditions, yet excessive crosslinking may reduce conformability, limit oxygen and moisture exchange, and hinder cell infiltration or therapeutic diffusion. Similarly, strong wet adhesion is advantageous for stable fixation on exudative wound beds and prolonged local retention, but overly adhesive dressings may damage fragile neo-tissue during removal. Likewise, highly permeable or highly swollen networks may improve moisture balance and mass transport, but may also compromise mechanical stability and accelerate premature drug release. Therefore, the rational design of hydrogels for diabetic wounds should not focus on maximizing a single material property, but rather on balancing mechanical robustness, adhesiveness, permeability, degradability, and spatiotemporal release behavior according to wound pathology and healing stage.

At the same time, the gap between preclinical success and clinical translation remains substantial. Most hydrogel systems reported to date have been validated primarily in rodent wound models, which do not fully reproduce the chronicity, tissue scale, biomechanical loading, comorbidity burden, and heterogeneous infection status observed in human diabetic wounds. Clinical evidence is still limited, and high-quality randomized controlled trials directly comparing advanced hydrogel systems with current standard-of-care dressings remain insufficient. In addition, the translation of multifunctional or stimuli-responsive hydrogels faces important practical barriers, including scalable and cost-effective manufacturing, batch-to-batch reproducibility, sterilization compatibility, shelf stability, storage requirements, and ease of use in routine wound care. These challenges become even more pronounced for hydrogels incorporating bioactive cells, exosomes, growth factors, nanozymes, or integrated sensing/electronic modules, because such systems may increase product complexity, production cost, and quality-control burden. Regulatory approval may also be more complicated than for conventional dressings, as many advanced hydrogel platforms fall at the interface of biomaterials, drug delivery systems, combination products, and digital medical devices, thereby requiring more rigorous evaluation of safety, efficacy, biocompatibility, degradation behavior, and device reliability. Therefore, claims regarding “clinical potential” should be interpreted with caution unless supported by robust translational evidence.

Overall, although no single hydrogel can fully address the complexity of diabetic wounds, the modular and highly tunable nature of these materials makes them particularly well-suited for multi-target intervention. Future progress should move beyond simple functional stacking toward pathology-matched and stage-adaptive hydrogel systems that dynamically coordinate antibacterial action, inflammation control, angiogenesis, and tissue remodeling throughout the healing process. Equally important, future studies should place greater emphasis on translationally relevant design criteria, including manufacturing feasibility, regulatory strategy, cost-effectiveness, long-term safety, and clinical validation in well-designed human trials. With continued advances in biomaterials engineering and more rigorous translational evaluation, hydrogel-based dressings may become increasingly important components of precision therapy for diabetic wounds.

## Data Availability

No new data were created or analyzed in this study.

## Appendix A

**Table A1:** Summary of hydrogel-based strategies for diabetic wound healing.

Categories	Hydrogels	Models	Outcome	References
NIR stimuli-responsive hydrogel	LIN@PG@PDA composite hydrogel (GelMA/PNIPAM semi-IPN + PDA nanoparticles; linagliptin-loaded; 405 nm photocrosslinking; 808 nm NIR trigger)	HUVECs; STZ-induced diabetic SD rat full-thickness wound	In vitro:• NIR-triggered volume shrinkage enables controllable drug release • Enhances endothelial migration/angiogenesis (↑VEGF) In vivo:• Accelerates diabetic wound closure with increased neovascularization (↑CD31/α-SMA)	[9]
ROS-sensitive + photothermal antibacterial + local glycemia management hydrogel	ACG gels (ROS-sensitive PVA/TSPBA hydrogel co-loaded with Au–CeO2 dumbbells and GOx)	Diabetic BALB/c mouse wounds; antibacterial assays (*S. aureus*/*E. coli*); *S. aureus*–infected diabetic wound model	In vitro:• ROS scavenging improves inflammatory milieu and promotes M1 → M2 polarization • ROS-triggered GOx release lowers local glucose In vivo:• NIR photothermal action strengthens antibacterial efficacy and improves healing in infected diabetic wounds	[12]
Zwitterionic hydrogel with sustained delivery	Photopolymerized ZW hydrogel (SBMA/HEMA; optionally loaded with CNP–miR146a)	db/db mice full-thickness wounds	In vivo:• Supports sustained release of nanoparticles/proteins • ZW gel alone improves closure (higher SBMA fraction heals faster) • 40:60 SBMA:HEMA + CNP–miR146a achieves >90% closure by day 10; full closure around days 11–13	[18]
pH/ROS dual-responsive hydrogel with spatiotemporal delivery	POD/CE glycopeptide hydrogel (PBA-grafted oxidized dextran + caffeic acid–grafted ε-polylysine; Schiff base + boronic ester dynamic bonds; DS + MIC@MF embedded)	RAW264.7; L929; HUVECs; STZ-diabetic SD rat full-thickness wound with *S. aureus* infection	In vitro:• pH/ROS-triggered network dissociation enables DS fast release and MF sustained release • Intrinsic antibacterial/antioxidant effects reduce infection and oxidative stress In vivo:• Suppresses inflammation (↓TNF-α/IL-6, ↑IL-10) and promotes angiogenesis (↑VEGF/CD31/HIF-1α), accelerating infected diabetic wound repair	[21]
ROS-responsive nanofibrous dressing (multidrug + AuNRs)	PUDF electrospun nanofibers (ROS-responsive Se-containing polyurethane; loaded with Def + Indo + AuNRs)	L929; RAW264.7 (LPS); HUVECs (scratch/transwell); SD rat full-thickness wound; MRSA-infected wound	In vitro:• ROS accelerates drug release and provides strong radical scavenging In vivo:• Reduces MRSA burden and speeds wound closure (near-healed by day 14) • Immunomodulation (↓TNF-α/IL-6, ↑IL-10) and enhanced angiogenesis (↑HIF-1α/VEGF/CD31) with improved collagen organization and reduced scarring	[22]
Mild photothermal antibacterial hydrogel (NIR)	COG-Z@P200 hydrogel (CMCS/Gel/OSA dual-crosslinked; ZIF-8@PDA nanoparticles; 808 nm NIR mild PTT ~40–45 °C)	L929; MRSA/*E. coli*; STZ-diabetic C57BL/6 mouse MRSA-infected full-thickness wound	In vitro:• >99.5% MRSA killing under NIR (mild PTT + Zn^2+^ synergy) • Suppresses MRSA metabolism/virulence, weakening biofilm/pathogenicity In vivo:• Decreases ROS, promotes M2 polarization (Arg-1↑/TNF-α↓), enhances angiogenesis (VEGF/CD31↑) and collagen deposition, accelerating infected diabetic wound healing	[25]
Photo-controlled thermosensitive multifunctional hydrogel (NIR)	A-MPDA/Rh@T-Gel (thermosensitive gel with arginine-doped mesoporous PDA nanoparticles + rhein; catalyzes H_2_O_2_ → O_2_; 808 nm NIR-responsive)	NIH/3T3; HUVECs; RAW264.7; L929; STZ-diabetic BALB/c mouse *S. aureus*–infected wound	In vitro:• NIR mild PTT + rhein yields strong antibacterial activity (*S. aureus*/*E. coli*; reduced in vivo bacterial load) • Antioxidant ROS reduction and oxygen generation alleviate hypoxia (NIR-enhanced, repeatable) In vivo:• Suppresses inflammation and promotes migration/angiogenesis (CD31↑) and collagen deposition, accelerating infected diabetic wound closure	[29]
NIR photothermal hydrogel for growth factor delivery + antibacterial	PEGDA-GDY/G@TGF-β hydrogel (PEGDA matrix with GDY/gelatin core–shell nanocarrier loaded with TGF-β; NIR “covered → uncovered” release)	HDFs; HUVECs; bacteria (*S. aureus*, *E. coli*, P. aeruginosa, MRSA); STZ-diabetic C57BL/6 mouse full-thickness wound (daily NIR 30 s)	In vitro:• NIR enables ON/OFF programmable TGF-β release (higher cumulative release vs. no NIR) • Low-temperature PTT + GDY-mediated damage provides broad-spectrum antibacterial activity (incl. MRSA, P. aeruginosa) In vivo:• Enhances cell migration and angiogenesis (CD31↑), improving wound closure and tissue regeneration in vivo	[30]
Intrinsic antibiofilm + antioxidative hydrogel dressing (no irradiation)	PPN dressing (PEG network tethered cationic polyimidazolium, PIM-Mal, + N-acetylcysteine; film and Alg-PPN fiber forms)	Diabetic mouse infected wound biofilm models (MRSA or carbapenem-resistant P. aeruginosa); 3D ex vivo human skin equivalent	In vivo:• >3-log reduction (>99.9%) against MDR pathogens/biofilms, outperforming silver dressings • Faster in vivo healing (MRSA diabetic wounds ~fully closed by ~day 12; controls/silver not closed by 2 weeks) • Reduces inflammatory burden and proteases (↓CD11b^+^ infiltration, ↓pro-MMP9) and increases pro-repair factors (↑VEGF-A/PDGF-BB/FGF-2/EGF); improves granulation and collagen maturation (higher type I collagen fraction)	[31]
Chitosan-based immunomodulatory hydrogel dressing	PAAc/CFCS-vanillin hydrogel (catechol-functional chitosan + poly(acrylic acid); CFMC-Ag nanoparticles + vanillin; Schiff base; pH-dependent release)	3T3, HUVECs, RAW264.7; *S. aureus*/*E. coli* antibacterial; infected full-thickness wounds and diabetic wound models in rats	In vivo:• Broad antibacterial activity and faster closure in infected wounds • ROS scavenging reduces oxidative damage markers (4-HNE, 8-OHdG) in diabetic wounds • Anti-inflammatory immunoregulation (↓TNF-α/IL-6, ↑IL-4/IL-10) with M1 → M2 polarization (CD206↑) and enhanced angiogenesis (CD31/VEGF↑), accelerating diabetic wound repair	[37]
All-natural immunomodulatory bioadhesive hydrogel	FGMA/FG/PA hydrogel (photo-crosslinked FGMA with physically entangled FG/PA; PA = protocatechuic aldehyde; Schiff base)	RAW264.7; L929; HUVECs; *S. aureus*–infected wounds (SD rats); STZ-diabetic SD rat full-thickness wound	In vitro:• Intrinsic bioadhesion, antibacterial activity, and ROS scavenging • Induces M1 → M2 transition (↓TNF-α/IL-1β; ↑TGF-β/VEGF) without extra additives In vivo:• Promotes angiogenesis (↑CD31/α-SMA) and accelerates diabetic wound closure with improved collagen remodeling and appendage regeneration	[39]
Macrophage-modulating HA-based hydrogel	HA-PF (high-MW hyaluronic acid hydrogel loaded with paeoniflorin)	BMDMs; STZ-diabetic C57BL/6J mouse full-thickness wound; L929/HUVEC cytocompatibility	In vitro:• Drives M1 → M2 polarization (↓CD86/iNOS/TNF-α/IL-1β; ↑CD206/Arg-1/IL-10/TGF-β) In vivo:• Enhances angiogenesis and repair markers (↑CD31/VEGF/α-SMA/Collagen I) • Accelerates closure vs. blank HA and commercial INTRASITE gel, reducing inflammation and improving re-epithelialization/collagen deposition	[41]
Nanofiber–hydrogel composite dressing	P-4HC nanofiber hydrogel (PLA/4HC short nanofibers embedded in PVA/HHA hydrogel; 4HC 1.5–3 mg/mL)	L929; HUVECs; RAW264.7 under high-glucose; db/db mouse full-thickness wound (bFGF gel control)	In vitro:• Sustained release, high water retention, and good hemocompatibility • Promotes angiogenesis (↑HUVEC migration/tube formation; ↑CD31/VEGF-A) and alleviates hypoxia (↓HIF-1α) In vivo:• Drives M1 → M2 (↓CD86/iNOS/IL-6/IL-1β/TNF-α; ↑CD206/Arg-1/IL-4/IL-10/VEGF), accelerating closure and collagen deposition (↑COL3A1); mechanism involves suppression of TLR/IL-17/TNF signaling	[42]
Biofactor-loaded GelMA hydrogel (immunomodulatory + pro-angiogenic)	GelMA–CGRP hydrogel (GelMA loaded with recombinant CGRP; 405 nm photocrosslinking; sustained release)	BMDMs, HUVECs, L929; *E. coli* inhibition;STZ + HFD-induced T2D C57BL/6J mouse full-thickness wound	In vitro:• Sustained CGRP release with good cytocompatibility and enhanced antioxidant capacity • Promotes M2 polarization via MDM2/p53/p21-related signaling (↑IL-10/TGF-β/VEGFA; ↓TNF-α/IL-6/IL-1β) • Enhances endothelial function (↑proliferation/migration; ↑VEGFA) and angiogenesis In vivo:• Accelerates diabetic wound closure with improved granulation, re-epithelialization, and collagen deposition (↑CD31/Ki67/α-SMA)	[43]
Self-healing drug-loaded hydrogel dressing	BP hydrogel (PVA–borax–puerarin; dynamic borate/boronic ester bonds; injectable, self-healing, ductile)	L929 biocompatibility; *S. aureus* antibacterial; STZ-diabetic SD rat chronic full-thickness wound	In vitro:• Excellent biocompatibility/hemocompatibility • Dose-dependent antibacterial activity against *S. aureus* In vivo:• Accelerates diabetic wound repair with improved re-epithelialization, neovascularization, and collagen organization • Mechanistically promotes M1 → M2 (iNOS↓, CD206↑), enhances angiogenesis (VEGFA/CD31↑), and collagen remodeling (COL-I/COL-III↑)	[44]
Nanozyme-modified antibacterial GelMA hydrogel (EC–macrophage crosstalk)	CeO2–Y@ZIF-8@Gel (CeO2–Y@ZIF-8 nanozyme/particles embedded in photocrosslinked GelMA; GelMA with quaternary ammonium salts for antibacterial activity)	HUVECs (ROS/mtROS, mitochondrial function, NLRP3 signaling); macrophages (cGAS–STING, M1/M2); infected diabetic mouse full-thickness wound	In vitro:• >99.99% killing against *S. aureus*/*E. coli* • Nanozyme scavenges ROS/mtROS and improves endothelial viability/proliferation • Suppresses endothelial NLRP3 activation and ox-mtDNA leakage • Downregulates macrophage cGAS–STING activation and promotes M1 → M2 (↓IL-6/IFN-γ, ↑IL-10) In vivo:• Fastest in vivo healing with enhanced angiogenesis (↑VEGF/CD31/α-SMA)	[46]
MMP-9-responsive exosome-releasing hydrogel	Exo@MRH (oxidized dextran + MMP-9-cleavable peptide + carboxymethyl chitosan hydrogel loaded with M2 macrophage-derived exosomes)	db/db mouse full-thickness wound; RAW264.7 polarization and exosome uptake; HDF scratch migration	In vitro:• Leverages elevated MMP-9 in diabetic wounds for inflammation-triggered degradation and on-demand exosome release • M2 exosomes promote M1 → M2 (↑CD206/Arg-1; ↓CD86/iNOS/TNF-α) In vivo:• Best in vivo closure with improved re-epithelialization and collagen deposition • Transcriptomics indicate downregulation of inflammatory pathways (NF-κB/IL-17/TNF/chemokine) and enhanced regeneration-related processes	[47]
Chemokine-sequestering + ROS-scavenging nanozyme hydrogel	Cu5.4O@Hep-PEG (starPEG–heparin hydrogel sequestering chemokines; loaded with ultrasmall Cu5.4O nanozymes)	chemokine binding (e.g., MCP-1/IL-8), fibroblast antioxidant assays, HUVEC proliferation/migration; leukocyte migration; acute wounds and STZ-diabetic BALB/c mouse wounds (Promogran^®^/Tegaderm controls)	In vitro:• Sequesters MCP-1/IL-8, reducing macrophage/neutrophil recruitment and infiltration • Broad ROS scavenging alleviates oxidative stress and lowers inflammatory mediators • Promotes angiogenesis and tissue regeneration (↑VEGF/CD31) In vivo:• Significantly accelerates closure in acute and diabetic wounds, outperforming Promogran^®^	[51]
ROS-responsive peptide-incorporated hydrogel	G/D-CuP (PVA crosslinked via ROS-responsive EBPBA phenylboronic ester; dimeric copper peptide D-CuP released under ROS)	RAW264.7; NIH/3T3/L929; HUVECs; STZ + HFD T2D C57BL/6 mouse full-thickness wounds (including infected wounds)	In vitro:• ROS scavenging with ROS-triggered D-CuP release (accelerated release under H_2_O_2_) • Promotes M1 → M2 and reduces inflammation (↓TNF-α/IL-6, ↑IL-10) • Enhances angiogenesis and cell proliferation/migration (↑VEGF, ↑CD31, ↑Ki67; improved tube formation) In vivo:• Accelerates diabetic wound repair, achieving high closure in infected wounds	[56]
Transparent conductive adhesive hydrogel patch for DFU + glucose monitoring	P(Py-TA)/CHA conductive patch (PTA-doped PPy nanofibrils in P(AM-Aa) network; MPBA for glucose responsiveness)	STZ-diabetic SD rat full-thickness wound; in vivo hemostasis models (liver puncture/incision, tail amputation); wound-site glucose sensing	In vitro:• High optical transparency (up to 93.6%) enables visual monitoring • Rapid hemostasis with reduced blood loss In vivo:• Accelerates diabetic wound closure with improved granulation, collagen deposition, angiogenesis (CD31↑), and re-epithelialization (K14↑) • Real-time glucose monitoring via linear resistance response to glucose (20–200 mM)	[66]
Self-monitoring + photothermal antibacterial hydrogel dressing (NIR)	Au/AgNDs@Gel (Au/Ag nanodots embedded in PVA hydrogel)	Type II diabetic rat bacteria-infected full-thickness wound with 808 nm NIR; in vitro antibacterial vs. *S. aureus*/*E. coli*	In vitro:• Synergistic PTT + Ag yields near-complete bacterial eradication under NIR In vivo:• Promotes infected diabetic wound healing (re-epithelialization and collagen deposition) • Anti-inflammatory (↓IL-6/TNF-α/IL-1β; ↑IL-4) and pro-angiogenic (↑VEGF/CD31) effects • Fluorescence decay reflects nanodot release, providing a visual cue for dressing replacement	[67]
Hypoxia-induced exosome-loaded in situ forming hydrogel	PB-Exo hydrogel (HB-PEG acrylate + HA-SH via thiol–ene Michael addition; in situ gelation)	HDF scratch; HUVEC tube formation/uptake; STZ-diabetic rat excisional wound	In vitro:• Rapid in situ gelation (~3 min) without initiators/toxic byproducts; sustained exosome release (~15 days) • Improves fibroblast migration and endothelial tube formation; prolongs exosome uptake/effect In vivo:• Accelerates diabetic wound closure with reduced inflammation (TNF-α↓) and increased mature vessels (CD31/α-SMA↑) • Increases bFGF and TGF-β1/β3, elevating TGF-β3/TGF-β1 ratio (anti-scar tendency)	[71]
Sprayable exosome-loaded photocrosslinkable ADM-based hydrogel	Exo@AMCN (methacrylated acellular dermal matrix; hUCMSC-Exo + β-CD–borneol; 405 nm photocrosslinking; curing time tunes degradation/release over ~2–7 days)	HaCaT scratch; HUVEC tube formation/VEGF; RAW264.7 (LPS); STZ-diabetic BALB/c mouse full-thickness wound	In vitro:• Programmable release window via curing time (≈2/5/7 days) • Multifunctional: antibacterial (>85% inhibition vs. *S. aureus*/*E. coli*), intracellular ROS reduction, enhanced migration/angiogenesis, and anti-inflammatory polarization In vivo:• Markedly accelerates in vivo healing with increased collagen deposition and neovascularization (CD31/VEGF↑), reduced inflammation (iNOS↓, CD206↑)	[72]
Exosome-coated oxygen nanobubble-laden self-healing hydrogel	EBO-Gel (ADSC exosome–coated BSA oxygen nanobubbles embedded in PVA/gelatin + borax dynamic hydrogel)	Fibroblasts under hypoxia; HUVEC tube formation; hemolysis and rat liver hemorrhage hemostasis; SD rat full-thickness wound	In vitro:• Oxygen supply alleviates hypoxia and reduces hypoxic signaling • Scavenges ROS/H_2_O_2_ via borate bonds and exosome-associated antioxidation • Enhances intracellular exosome cargo delivery under hypoxia by mitigating hypoxia-driven endocytic recycling • Promotes fibroblast proliferation/migration and angiogenesis In vivo:• Accelerates in vivo closure with improved collagen organization, increased CD31^+^ vessels, reduced ROS, M2 shift (CD206↑/CD86↓), and lower scar index	[73]
Sequentially triggered triple-responsive hydrogel (photothermal + glucose/pH)	APMDCG alginate hydrogel (ALG-PBA + Cu-PDA + metformin + DFO; Ca^2+^ crosslinked; PBA confers glucose/pH response; PDA enables NIR PTT)	RAW264.7 (LPS); HUVECs (MGO + hypoxia); STZ-diabetic C57BL/6 mouse *S. aureus*–infected wound	In vitro:• Infection-stage antibacterial: 808 nm NIR heating (~55 °C) plus Cu^2+^ release for strong bacterial killing • Hyperglycemia/acidity-triggered release of MET/DFO/Cu^2+^ (faster at high glucose and pH 5.0) • MET suppresses NLRP3/Caspase-1/GSDMD/IL-1β inflammatory axis • DFO activates HIF-1α/VEGF to improve endothelial function and angiogenesis In vivo:• Best in vivo repair with lowest residual area, reduced bacterial colonization, increased collagen deposition and skin thickness	[76]
ROS-responsive nanocomposite hydrogel (quercetin)	GelMC/PVA-UIO-66-NH2@Que (borate-bond ROS-responsive hydrogel incorporating UIO-66-NH2@Que; UV photocrosslinking)	L929; RAW264.7 (LPS); HUVECs (conditioned medium assays); STZ-diabetic SD rat full-thickness wound	In vitro:• ROS-triggered on-demand quercetin release • Reduces intracellular ROS and promotes M1 → M2 (↓CD86/IL-6/TNF-α; ↑CD206/IL-10) • Enhances angiogenesis via macrophage modulation (↑HUVEC migration/tube formation; ↑VEGFA) In vivo:• Accelerates diabetic wound healing with increased vascular maturation (↑CD31/α-SMA)	[78]
ECM-inspired multifunctional nanofibrous hydrogel (MR-mediated immunomodulation)	GM-Pgel/GM-P hydrogel (glucomannan + polypeptides; Schiff base + H-bonding; multifunctional peptides: AMP antibacterial, CAP antioxidant/adhesive, PAP pro-angiogenic; hierarchical nanofibrous porous network; injectable/self-healing/biodegradable)	NIH/3T3, HUVECs, HaCaT; RAW264.7 (M1/M2); antibacterial vs. MRSA/*E. coli*; STZ-diabetic SD rat MRSA-infected wound	In vitro:• Potent antibacterial activity (>95% sterilization vs. MRSA/*E. coli*) via membrane disruption • Strong ROS scavenging (>80% intracellular ROS reduction) • Mannose receptor activation drives M2 polarization (CD206↑, CD86↓) and reduces pro-inflammatory cytokines while increasing IL-10/TGF-β In vivo:• Promotes angiogenesis (↑CD31/α-SMA) and accelerates infected diabetic wound closure with improved epidermal regeneration and collagen organization	[79]
AuNP-based antibacterial hydrogel for drug-resistant infection	Gel/CS-AuNPs hydrogel (gelatin/SA matrix with chitosan-functionalized AuNPs; Gel/CS-Au25 optimal)	*E. coli*, *S. aureus*, MRSA; NIH3T3 cytocompatibility; type II diabetic SD rat MRSA-infected full-thickness wound	In vitro:• Broad-spectrum antibacterial activity including MRSA; dose-dependent, Gel/CS-Au25 best overall In vivo:• Accelerates infected diabetic wound closure (day 15 residual area ~3.1% for Gel/CS-Au25) • Reduces inflammation (↓TNF-α, ↑IL-10), enhances angiogenesis (↑CD31), and improves collagen deposition/remodeling and re-epithelialization	[83]
